# RvD2 mitigates TNFɑ-Induced mitochondrial reactive oxygen species through NRF2 signaling in placental trophoblasts

**DOI:** 10.3389/fphys.2025.1547940

**Published:** 2025-04-02

**Authors:** Taija Hahka, Deekshika Sekar, Prakash Kumar Sahoo, Aiswariya Ravi, Colman Freel, Chandan Krishnamoorthy, Sankar Ramamurthy, Rebekah Rapoza, Rebecca Drakowski, Anum Akbar, Matt VanOrmer, Melissa Thoene, Corrine K. Hanson, Tara Nordgren, Sathish Kumar Natarajan, Ann Anderson Berry

**Affiliations:** ^1^ Department of Cellular and Integrative Physiology, University of Nebraska Medical Center, Omaha, NE, United States; ^2^ Department of Nutrition and Health Sciences, University of Nebraska at Lincoln, Lincoln, NE, United States; ^3^ Department of Pediatrics, University of Nebraska Medical Center, Omaha, NE, United States; ^4^ College of Allied Health Professions, University of Nebraska Medical Center, Omaha, NE, United States; ^5^ Department of Environmental and Radiological Health Sciences, Colorado State University, Fort Collins, CO, United States

**Keywords:** hypertensive disorders of pregnancy, omega-3 fatty acids, oxidative stress, glutathione, metabolism, superoxide, antioxidant, lipid mediators

## Abstract

**Introduction:**

Hypertensive disorders of pregnancy (HDP) are marked by elevated levels of TNFα, which increases reactive oxygen species (ROS) and disrupts metabolism of trophoblasts. Resolvin D2 (RvD2), an omega‐3 fatty acid-derived lipid mediator, is known to resolve inflammation, but its role in protecting trophoblasts by promoting antioxidant responses to alleviate ROS remains unclear. Nuclear translocation of nuclear factor erythroid 2‐related factor 2 (NRF2) controls cellular defense mechanisms against oxidative stress and helps with the maintenance of cellular redox homeostasis. Upon translocation to nucleus, NRF2 activates the antioxidant response element (ARE), inducing the expression of genes that can mitigate ROS. Hence, we hypothesized that RvD2 activates NRF2 and prevents TNFα‐induced mitochondrial dysfunction in trophoblasts.

**Methods:**

We investigated RvD2’s potential protective mechanisms against TNFα‐induced oxidative stress in trophoblasts by pretreating JEG cells with 100 nM RvD2, followed by exposure to 50 or 100 ng/mL TNFα.

**Results:**

We also observed that placental TNFα levels were elevated, while NRF2 protein levels were reduced in human HDP placental tissues compared to normotensive placentas. We demonstrate that RvD2 alone enhances NRF2 nuclear translocation, increases glutathione levels and mitochondrial function, and reduces mitochondrial ROS. In contrast, TNFα alone decreases nuclear NRF2 levels, increases mitochondrial ROS and oxygen consumption rates, and impairs migration. Notably, pretreatment of RvD2 before TNFα exposure protects against mitochondrial ROS, increases NRF2 levels, and restores mitochondrial oxygen consumption rates in trophoblasts.

**Discussion:**

These findings demonstrate that RvD2 functions as a positive regulator of endogenous antioxidant properties by enhancing NRF2 levels and mitigating mitochondrial ROS in placental trophoblasts.

## Graphical Abstract

**Figure:**
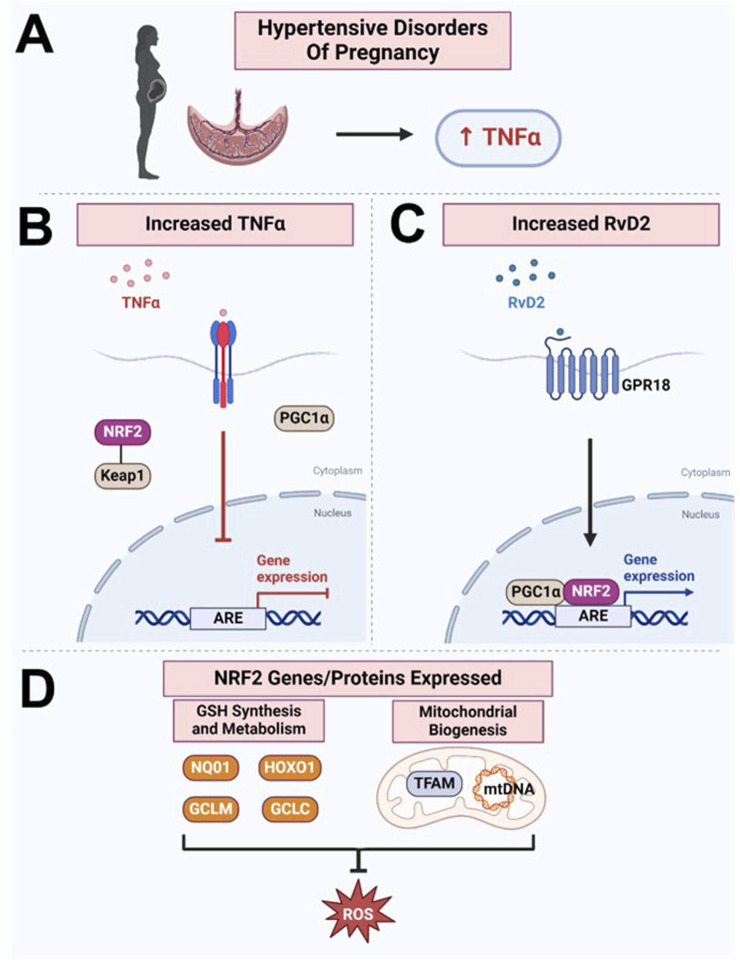
Paper Schematic: Our study found that **(A)** TNFα levels were increased in the hypertensive human placenta, while **(B)** NRF2 levels are decreased, suggesting a disruption in this protective mechanism against oxidative stress. **(C)** We discovered that RvD2 can target the NRF2 pathway, facilitating upregulation of the transcription factor NRF2, which, together with PGC1ɑ, binds to the ARE, **(D)** promoting the expression of genes and proteins responsible for glutathione synthesis and metabolism and enhancing the cell’s antioxidant capacity. Additionally, RvD2 activation may promote mitochondrial biogenesis by increasing TFAM and mtDNA, collectively inhibiting ROS production. However, the elevated TNFα in hypertensive placenta may counteract these beneficial effects by inhibiting NRF2’s and PGC1a’s abilities to bind to the ARE. Image created with BioRender.com.

## 1 Introduction

Hypertensive disorders of pregnancy (HDP), including chronic hypertension, gestational hypertension, preeclampsia, and eclampsia, impact 10% of pregnant individuals worldwide (Hahka et al., 2024). HDP is characterized by poor placental trophoblast invasion and migration, resulting in maternal inflammation and oxidative stress, which impair nutrient and oxygen transfer to the fetus (Hahka et al., 2024; Sultana et al., 2023). Elevated pro-inflammatory cytokines, especially tumor necrosis factor-ɑ (TNFɑ), induce reactive oxygen species (ROS), leading to mitochondrial dysfunction and reduced metabolic function in placental trophoblasts, worsening HDP (Marin et al., 2020; Smith et al., 2021; Dos Passos Junior et al., 2021; Wang and Walsh, 1996). Inhibition of oxidative damage *via* targeted therapies may offer a promising approach for the development of novel treatments to prevent HDP.

Resolution of inflammation is a complex, highly regulated process involving omega-3 fatty acid-derived specialized pro-resolving lipid mediators (SPMs), such as resolvins, protectins, maresins, and lipoxins (Thompson et al., 2022; Stark et al., 2013). Resolvins (Rv) are divided into E- and D-series including RvE1-RvE4 and RvD1-RvD6 (Thompson et al., 2022). Our published data showed that RvD2 levels are significantly higher in maternal blood at delivery compared to cord blood, highlighting the placenta as a key SPM action site (Nordgren et al., 2019). We have also earlier established that RvD2 enhances plasma membrane translocation of GPR18 in placental trophoblasts. GPR18 activation is associated with promoting vasodilation and contributing to blood pressure reduction in rodent models (Matouk et al., 2017; Penumarti and Abdel-Rahman, 2014; Ulu et al., 2019). Furthermore, we have confirmed that treatment of RvD2 can increase the production of anti-inflammatory cytokine, IL-10 in TNFα-treated cells (Ulu et al., 2019). Studies have reported that RvD2 promotes tissue healing, modulates immune responses by limiting M2 macrophage polarization (Pope et al., 2016), improves cardiovascular function (Diaz Del Campo et al., 2023), and displays analgesic properties (Perna et al., 2021). Although less is known about the impact of RvD2 during pregnancy, omega-3 fatty acids, the substrate for RvD2, are crucial for fetal brain and eye development and are considered safe during pregnancy (Thompson et al., 2019).

Extensive research supports anti-inflammatory and pro-repair effects of RvD2, but its role in promoting or regulating antioxidant production, including the putative mechanisms involved, remains unclear. Oxidative stress occurs when ROS overwhelm antioxidant defenses, disrupting cellular homeostasis. Introducing RvD2 as a positive regulator of endogenous antioxidants could restore balance and mitigate oxidative damage, particularly in HDP, where oxidative imbalance impairs placental development (Hahka et al., 2024; Sultana et al., 2023). However, RvD2’s ability to regulate antioxidants are not well understood, with only two studies addressing its effects. Chen et al. (2020) found RvD2 increased glutathione and superoxide dismutase in diabetic mouse retinas, and Zhang et al. (2024) showed it reduced oxidative stress and protected the blood-brain barrier in brain-injured animals. However, neither study focused on placental trophoblasts or key antioxidant transcription factors. Studies on nuclear factor erythroid 2-related factor 2 (NRF2), the master regulator of antioxidant responses, are needed, as it counteracts oxidative stress by activating downstream pathways (Dodson et al., 2019; Lin et al., 2023; Bellezza et al., 2018; Ulasov et al., 2022). Upon activation, NRF2 dissociates from kelch-like ECH-associated protein 1 (KEAP1), translocate to the nucleus, and binds to the antioxidant response element (ARE) (Dodson et al., 2019; Lin et al., 2023; Bellezza et al., 2018; Ulasov et al., 2022). Literature suggests that chronic oxidative stress can impair NRF2 activation, diminishing antioxidant defenses and mitochondrial function (Bellezza et al., 2018; Shetty et al., 2017; Kim and Vaziri, 2010; Chen et al., 2015; van Horssen et al., 2010). Peroxisome proliferator-activated receptor gamma coactivator 1-alpha (PGC1α) is known to interact with NRF2/NRF1 to upregulate the expression of genes involved in mitochondrial biogenesis. One key downstream target of NRF2/PGC1ɑ is mitochondrial transcription factor A (TFAM), which is essential for mitochondrial DNA (mtDNA) replication, transcription, and maintenance, ensuring proper mitochondrial function (Picca and Lezza, 2015; Gureev et al., 2019). However, the signaling pathways through which RvD2 regulates NRF2 and PGC1α activation and influences mitochondrial function in placental trophoblasts remain unexplored.

To fill this gap, the present study investigated if RvD2 protects human placental trophoblasts from TNFɑ-induced oxidative damage and explored the underlying mechanisms involved. Here, we show that RvD2 protects human placental trophoblasts from TNFɑ-induced oxidative stress by activating the NRF2 signaling pathway and inducing the expression of PGC1α and TFAM, benefiting mitochondrial function.

## 2 Methods

### 2.1 Human placenta subjects

This study received ethical approval from the University of Nebraska Medical Center’s IRB (IRB #0112-15-EP). Subjects were enrolled at delivery, with written consent obtained from the mothers. Term placental cross-sections were obtained and stored in −80°C. Cotyledons were selected from random locations on the placenta. Each section was free of major calcification or blood clots. Decidua and amniotic membranes were removed. Participants included women aged 19 to 39 admitted to an academic medical center’s (Nebraska Medicine, Omaha, NE, United States) Labor and Delivery unit from 2020-2021. Exclusion criteria included conditions affecting nutrient metabolism (liver, gastrointestinal, or kidney diseases), congenital abnormalities, inborn metabolic errors, and infants classified as wards of the state. This study used the American College of Obstetricians and Gynecologists (ACOG) criteria for hypertensive disorders in pregnancy ACOG Practice Bulletin 222.

### 2.2 Cell culture

JEG-3 cells (ATCC, VA, United States), which are a hypertriploid, clonally derived human cell line with epithelial morphology originating from choriocarcinoma (third-trimester placental trophoblast-like cells), were cultured in MEM (Corning, NY, United States) supplemented with 10% fetal bovine serum (Gibco, NY, United States) and plasmocin (InvivoGen, CA, United States). The cells were maintained at 37°C in a humidified 5% CO_2_ incubator and regularly passaged every 3–4 days, with no more than 14 passages to maintain morphology. In our manuscript, these trophoblast-like cells are referred to as trophoblasts.

### 2.3 Materials and treatment strategies

Resolvin D2 (RvD2, Cayman Chemical #10007279) was purchased from the manufacturer as 20 µg in 250 µL of 100% ethanol stored at −80°C. RvD2 was prepared by dissolving in ethanol for a stock concentration of 100 µM. For cell treatments, the RvD2 stock was diluted to appropriate concentrations in MEM plus 10% fetal bovine serum cell medium.

Tumor necrosis factor alpha (TNFɑ; Abcam, #ab9642), a lyophilized powder stored at −80°C, was reconstituted in ddH2O to a stock concentration of 1.0 mg/mL. For treatments, the stock solution was diluted to the required concentrations in MEM supplemented with 10% fetal bovine serum.

TNFɑ was added by replacing half of the medium to minimize cell disruption. TNFɑ groups represent the time points of TNFɑ addition to RvD2-treated cells (e.g., 5 h of TNFɑ followed 16 h of RvD2). For RvD2 and Vehicle groups, the same process was followed, using an equivalent amount of ethanol for the Vehicle group. Vehicle treatment consisted of <1% ethanol in FBS-containing medium.

MG132, a proteasome inhibitor (carbobenzoxy-Leu-Leu-leucinal) was purchased from Sigma (MG132, Sigma, #M8699) and prepared in dimethyl sulfoxide.

The time frames for studying proteins, metabolic processes, reactive oxygen species, mRNA, and cellular migrations differed due to the unique kinetics and processes associated with each aspect. The treatment details and time frames for each experiment are described in each methodological section.

### 2.4 Nuclear, cytoplasmic, and mitochondrial isolation

Cells were treated at 70%–80% confluences and then were washed with phosphate-buffered saline (PBS, 1X) and then Buffer A [10 mM HEPES, 10 mM KCl, 0.1 mM EDTA, 0.1 mM DTT, and 0.5% nonidet-P40 substitute (MilliporeSigma) supplemented with cOmplete Protease Inhibitor Cocktail™ (Roche, #05892791001)] was applied to the cells. The cells were scraped and incubated on ice for 10 min. After incubation, the lysate was centrifuged at 15,000 × *g* for 3 min at 4°C, and the cytosolic proteins in the supernatant were collected and stored at −80°C for future use. The nuclear pellet was resuspended in Buffer B (20 mM HEPES, 0.4 M NaCl, 1 mM EDTA, 0.05 mM DTT, 10% glycerol, and cOmplete Protease Inhibitor Cocktail™) and incubated on ice with intermittent vortexing for 40 min. Nuclear proteins were isolated by centrifugation at 15,000 × *g* for 10 min at 4°C, and the supernatant fraction was stored at −80°C for subsequent protein quantification and immunoblot analysis.

To isolate mitochondria, JEG-3 cells were trypsinized and washed twice *via* centrifugation in ice-cold PBS. The cell pellets were resuspended in a buffer containing 5 mM Tris, 0.25 M sucrose, 1 mM EDTA, and Halt™ protease and phosphatase inhibitor cocktail (pH 7.4). Cells were homogenized on ice using a Dounce homogenizer for 1 min, then centrifuged at 600 *g* for 10 min at 4°C. The pellet, containing cell debris, was discarded. The supernatant was further centrifuged at 17,000 × *g* for 15 min at 4°C to obtain mitochondria. The mitochondrial pellet was resuspended in suspension buffer including 5 mM Tris, 0.25 M sucrose (pH 7.4), and centrifuged again at 17,000 × *g* for 15 min at 4°C, followed by two additional washes in suspension buffer. The final mitochondrial pellets were stored in suspension buffer and kept frozen at −80°C for subsequent protein quantification and immunoblot analysis.

### 2.5 Live-cell imaging of mitochondrial reactive oxygen species

JEG-3 cells were cultured to 70%–80% confluency on MatTek 35 mm glass-bottom dishes (P35G-1.5-20-C) and pre-treated with Vehicle, RvD2, or Mitoquinone mesylate (MitoQ, TargetMOI Chemicals #845959-50-4) at 16 h prior to imaging. Cells were incubated in MitoSOX™ (Invitrogen, MA, United States #M36008) per manufacturer’s recommendations. TNFɑ treatments were added during live cell imaging. To minimize disturbance, half of the medium was removed and replaced with fresh medium containing TNFα, resulting in a final concentration of 100 ng/mL. Images were captured before and after addition of TNFɑ or medium. Confocal imaging was conducted using a Nikon A1R-Ti2 Confocal Laser Scanning Microscope, equipped with a Plan Apo 60 × 1.40 NA oil-immersion objective (0.17 WD, 0.13) for a total magnification of ×600. Time-lapse imaging was performed at 1-min intervals over a duration of 15 min, utilizing a Tokai Hit INU Environmental Incubation Chamber set to 37°C and integrated with the Nikon Ti2 Inverted Fluorescent Microscope. Non-sequential imaging was employed, with the 488 nm laser (green channel) used to excite MitoSOX™, and emission was collected in the 500–550 nm range. Emission from MitoSOX™ was further analyzed in the red channel, utilizing the 560 nm laser for excitation and the 570–620 nm range for emission collection. All images were acquired using Nikon NIS Elements software and images were further processed using ImageJ software (National Institute of Health).

### 2.6 Immunofluorescence imaging of NRF2 nuclear translocation

JEG-3 cells were cultured to 70%–80% confluency on collagen-coated coverslips and subjected to treatments of RvD2, TNFɑ, or RvD2 + TNFɑ for 8, 16, or 24 h. Following different time points, cells were rinsed twice with warm PBS and fixed in 3% paraformaldehyde (Electron Microscopy Sciences, PA, United States) in PBS containing 100 mM PIPES, 3 mM MgSO_4_, and 1 mM EDTA for 20 min at 37°C. The cells were then washed three times with PBS and gently permeabilized at room temperature for 5 min with 0.3% Tween 20 in PBS. After permeabilization, the cells were washed with PBS and blocked for 60 min at 37°C in a solution of PBS containing 5% glycerol, 5% goat serum, and 0.01% sodium azide. An incubation with NRF2 primary antibody was performed in this buffer at a dilution of 1:100 and left overnight at 4°C. The following day, cells were washed three times with PBS and incubated with Alexa Flour-conjugated secondary antibodies (Invitrogen, MA, United States) for 60 min at 37°C. Afterward, cells were washed once with PBS, once with deionized water, and counterstained with DAPI. Following two additional washes with PBS, the cells were mounted onto microscope slides using Fluoromount-G (Electron Microscopy Sciences, PA, United States). Images were captured using a Nikon AIR-Ti2 Confocal Laser Scanning Microscope with a Plan Apo 60 × 1.40 NA oil-immersion objective (0.17 WD, 0.13), and further processed using ImageJ. In ImageJ, the image channels were split and converted to binary format to eliminate color discrepancies and biases. The relative intensity ratios between the channels were then calculated and compared.

### 2.7 Semi-quantitative protein analysis using immunoblots

Cells were pre-treated with RvD2 or Vehicle for 16 h, followed by the addition of TNFɑ, and then incubated for an additional 5 or 10 h, resulting in total treatment times of 21 or 26 h, labeled as “16 + 5” or “16 + 10” hours. Based on our preliminary findings, we have observed that 5- or 10-h TNFα treatment durations effectively captures both early and late cellular responses to inflammatory and metabolic stimuli (bioenergetic changes). A 5-h incubation allows for the analysis of initial signaling events and early gene expression changes, while a 10-h treatment provides insight into sustained activation and downstream functional effects such as changes in protein expression. We have measured bioenergetic changes across various time frames and pretreatment conditions. However, 5-h TNFα exposure showed dramatic changes in oxygen consumption rate.

Following treatments, cells were washed once with ice-cold PBS and then scraped from the plate in cell lysis buffer (50 mM Tris, pH 7.4, 150 mM NaCl, 1 mM EDTA, 1 mM DTT, 1 mM Na_3_VO_4_, 1 mM PMSF, 100 mM NaF, and 1% Triton X-100) supplemented with cOmplete Protease Inhibitor Cocktail™.

For human tissues, cross-sectioned placentas without attached decidua were homogenized in lysis buffer containing cOmplete Protease Inhibitor Cocktail™. Lysates of tissues or cells were incubated on ice for 30 min to ensure proper lysis, then centrifuged at 14,000 × *g* for 20 min at 4°C. Clear supernatant fraction, containing protein, was collected for further analysis. Protein concentrations were measured using the Pierce 660 nm protein assay reagent (Thermo Fisher, #22660), based on a modified Lowry method (Waterborg and Matthews, 1994). A total of 20–30 µg of protein per sample was resolved on a 10% or 4%–12% gradient SDS-polyacrylamide gels and transferred onto nitrocellulose membranes (Bio-Rad, CA, United States) using a Bio-Rad wet transfer system.

The membranes were incubated in 5% skim milk or 5% bovine serum albumin dissolved in Tris-buffered saline with 0.1% Tween 20 (TBS-T) to block non-specific binding. After blocking, membranes were incubated overnight at 4°C with primary antibodies listed in Table 1 (1:1,000 dilution). Following three washes with TBS-T, membranes were treated with HRP-conjugated secondary antibodies (1:5,000 dilution) for 2 h at room temperature. Protein bands were visualized using chemiluminescent substrates (Bio-Rad #170-5061; PerkinElmer #NEL104001; or Thermo Scientific #A38554) and imaged with the Bio-Rad ChemiDoc system.

**TABLE 1:** List of antibodies used.

Antibody	Company; catalog number
*β*-actin	Sigma; #A5441
GPR18	Thermo Fisher Scientific; PA5-23218
NRF2	Abcam; #AB137550-1001
PGC1ɑ	Thermo Fisher Scientific; #PA5-72948
SOD2	Cell Signaling Technology; #13194
TFAM	Thermo Fisher Scientific; #MA-516148
KEAP1	Cell Signaling Technology; #8047
VDAC	Cell Signaling Technology; #4661
HDAC1	Cell Signaling Technology; #34589
OXPHOS or ETC Complex Cocktail	Abcam; #AB110411

### 2.8 Relative mRNA quantification and mitochondrial DNA content measurements using real-time qPCR

Cells were pre-treated with RvD2 or Vehicle for 16 h, followed by the addition of TNFɑ and a further 5-h incubation, totaling 21 h of treatment, labeled as “16 + 5 h” Following treatments, JEG-3 cells were lysed directly in the wells or microcentrifuge tubes using TRIzol reagent (Invitrogen, MA, United States; #15596018), and total RNA was extracted following the manufacturer’s protocol.

The RNA was then quantified and assessed for purity using a Biotek Synergy plate reader with Gen5 software. To synthesize cDNA, 1 μg of total RNA was reverse transcribed using random hexamers, RNase OUT (Invitrogen, MA, United States), dNTPs, and Murine-MuLV reverse transcriptase (NEB, MA, United States). Quantitative real-time PCR (RT-qPCR) was carried out using Light Cycler 480 SYBR Green I Master mix (Roche, Basel, Switzerland; #04707516001) on a Bio-Rad CFX Connect system. The ∆ CT values for the mRNA of interest were quantified relative to 18S rRNA.

Mitochondrial DNA content was quantified by calculating the ratio of mtDNA (mND-1, mitochondrially encoded NADH dehydrogenase 1) to nuclear DNA (mPyruvate kinase) mRNA expression. The target genes and primer sequences (Integrated DNA Technology, IA, United States) are listed in Table 2.

**TABLE 2:** List of primers used.

Target gene	Primer sequence (forward, F; reverse, R)
18S rRNA	F: 5′-CGTTCTTAGTTGGTGGAGCG-3′ R: 5′-CGCTGAGCCAGTCAGTGTAG-3′
PGC1a	F: 5′-CCAAACCAACAACTTTATCTCTTCC-3′ R: 5′-CACACTTAAGGTGCGTTCAATAGTC-3′
NRF2	F: 5′-AGTGGATCTGCCAACTACTC-3′ R: 5′-CATCTACAAACGGGAATGTCTG-3′
TFAM	F: 5′-CGTTGGAGGGAACTTCCTGAT-3′ R: 5′-CCTGCCACTCCGCCCTATA-3′
TNFɑ	F: 5′-GGCAGTCAGATCATCTTCTCG-3′ R: 5′-GGTTTGCTACAACATGGGCTA-3′
GPR18	F: 5′-CCACCAAGAAGAGAACCAC-3′ R: 5′-GAAGGGCATAAAGCAGACG-3′
NQO1/NADPH	F: 5′-CCTGCCATTCTGAAAGGCTGGT-3′ R: 5′-GTGGTGATGGAAAGCACTGCCT-3′
KEAP1	F: 5′-CAACTTCGCTGAGCAGATTGGC-3′ R: 5′-TGATGAGGGTCACCAGTTGGCA-3′
HOXO1	F: 5′-CCAGGCAGAGAATGCTGAGTTC-3′ R: 5′-AAGACTGGGCTCTCCTTGTTGC-3′
GCLC	F: 5′-GGAAGTGGATGTGGACACCAGA-3′ R: 5′-GCTTGTAGTCAGGATGGTTTGCG-3′
GCLM	F: 5′-TCTTGCCTCCTGCTGTGTGATG-3′ R: 5′-TTGGAAACTTGCTTCAGAAAGCAG-3′
mPyruvate kinase	F: 5′-ACTGGCCGGTGTCATAGTGA-3′ R: 5′-TGTTGACCAGCCGTATGGATA-3′
mND-1	F: 5′-GGCTACATACAATTACGCAAAG-3′ R: 5′-TAGAATGGAGTAGACCGAAAGG-3′

### 2.9 Measuring mitochondrial bioenergetics or oxygen consumption rates

JEG-3 cells were seeded at 50,000 cells per well in a 24-well Seahorse XF24 Cell Culture Plate (Agilent # 100777-004). After 24 h, the cells were pre-treated with 100 nM RvD2 or Vehicle for 16 h followed by exposure with 100 ng/mL of TNFɑ for 5 h. Blank or background wells were used to measure the amount of medium to ensure consistency. The total treatment duration was 21 h, referred to as “16 + 5 h.”

The cartridge of the Seahorse Extracellular Flux Assay Kit (Agilent #100840-000) was hydrated overnight in a CO_2_-free incubator with XF calibrant (Agilent #100840-000), and prior to the assay, cells were carefully switched to Seahorse XFp medium (Agilent #103575-100) supplemented with 1 M glucose, 100 mM pyruvate, and 200 mM L-glutamine, but free of phenol red. The cells were then incubated in a CO_2_-free incubator for 1 h. Oligomycin (1 µM), FCCP (1 µM), and Rotenone/Antimycin A (0.5 µM each, final concentration) (Seahorse XF Cell Mito Stress Test Kit, Agilent #103015-100), were loaded into the ports of the cartridge. The Seahorse XFe24 Analyzer was used to measure the bioenergetic parameters of the cells.

After the run, cells were washed with ice-cold PBS, then lysed in lysis buffer (50 mM Tris, pH 7.4, 150 mM NaCl, 1 mM EDTA, 1 mM DTT, 1 mM Na_3_VO_4_, 1 mM PMSF, 100 mM NaF, and 1% Triton X-100) containing cOmplete Protease Inhibitor Cocktail™ before being wrapped tightly in parafilm and placed at −20°C overnight. The following day, after thawing the lysates, the cells were thoroughly mixed, and protein concentration was determined, and oxygen consumption rates (pmol/min) were normalized to mg/mL of protein.

### 2.10 Assessing mitochondrial function with flow cytometry

When the JEG-3 cells reached 70%-80% confluency they were pre-treated with Vehicle or RvD2 for 16 h. To minimize disturbance, half of the medium was removed and replaced with fresh medium containing TNFɑ, resulting in a final concentration of 100 ng/mL. The cells were incubated for an additional 1 h with TNFɑ or medium, resulting in a total treatment time of 17 h, labeled as “16 + 1 h.”

Cell viability was assessed using Zombie Violet™ (BioLegend, CA, United States, #423113) at a 1:1,000 dilution, incubated for 15 min at room temperature. JEG-3 cells positive for Zombie Violet™ were classified as functional or dysfunctional based on mitochondrial mass (MitoTracker™ Green, Invitrogen, MA, United States, #M7514) and membrane potential (MitoTracker™ Red CMXROS, Invitrogen, MA, United States, #M7512). MitoTracker™ Green and Red were prepared at 1 mM working concentrations in dimethylformamide and Dimethyl sulfoxide, respectively. These were further diluted to 100 nM working concentration each and co-incubated with the cells for 30 min at 37°C in a CO2 incubator. Following staining, cells were evaluated *via* flow cytometry using the Beckman Coulter CytoFLEX, with data processed in CytExpert software.

Cells with functional mitochondria were characterized as MitoTracker™ Red ^high (+)^ and MitoTracker™ Green ^high (+)^, denoted as “R + G+.” Cells with dysfunctional mitochondria were characterized as MitoTracker™ Red ^low (−)^ and MitoTracker™ Green ^high (+)^, denoted as “R-G+” further described by Monteiro et al. (2020).

### 2.11 Measurement of glutathione levels using glutathione detection assay kit

The Cellular Glutathione Detection Assay Kit (Cell Signaling Technology, Catalog #13859) was used to quantify reduced glutathione (GSH) concentrations, following the manufacturer’s instructions. Briefly, cells were seeded in a 96-well plate and when they reached 70%–80% confluency they were pre-treated with RvD2 for 16 h, followed by a 1-h treatment with TNFɑ, totaling 17 h and labeled as “16 + 1 h.”

### 2.12 Measurement of TNFɑ levels using an ELISA kit

TNFɑ levels were quantified using the Human TNF alpha ELISA kit (Invitrogen, #KAC1751) following the manufacturer’s protocol. Placental homogenates from human tissues, sectioned from random locations without attached decidua, were analyzed for the measurements.

### 2.13 Determining cell migration percentage using the scratch assay

JEG-3 cells were seeded at a density of 0.01 × 10^6^ cells per well in a 96-well plate and treated once they formed a monolayer and reached 80% confluency. Manually, one scratch was performed within each well using a 200 µL pipette tip. Immediately, the cells received treatments of vehicle, 10-100 nM of RvD2, or 10-50 nM of TNFɑ, or a combination of TNFɑ + RvD2. and then were imaged to mark the baseline of a 0-h and 24-h time point using BioTek Cytation 10 (Gen 5 Software), which is capable of capturing images from the same location.

The image was imported into ImageJ. The file type was changed to 8-bit (“Image” →“Type” →“8-bit”), and the image was smoothed five times (“Process” →“Smooth”). The contrast of the image was enhanced (“Process” →“Enhance Contrast … ”). The following options were selected: “Saturated pixels: 0.50%”, “Normalize.” The edges were found (“Process” →“Find Edges”) and the threshold was set (“Image” →“Adjust” →“Threshold”) by lowering the upper value of the threshold until the lateral areas with cells and the central area without cells were clearly defined.

Two borders were manually drawn on each side of the scratch using the Paintbrush Tool, and the areas outside the borders were filled in using the Flood Fill Tool. The particles of the image were analyzed (“Analyze” →“Analyze Particles … ”). The following options were selected: “Size (pixel^2): 100000-Infinity,” “Circularity: 0.00–1,000,” “Show: Nothing,” “Display: Results,” “Summarize,” “Add to Manager.” Then, the area was recorded. Percent wound closure was calculated using the following formula: $\left(1-\frac{final\;area}{initial\;area}\right)*100$ . Proofs were taken of each image by opening the original image in ImageJ and overlaying the ROI, or region of interest. (“Image” → “Overlay” → “From ROI Manager”).

### 2.14 MitoTracker green OD readings

When cells reached 70%–80% confluency, RvD2 was pretreated for 16 h, followed by a 5-h exposure to TNFα (for a total time of 21 h) as described earlier.

MitoTracker™ Green FM dye was reconstituted to a 1 mM stock solution in dimethylformamide and then diluted into medium to achieve a final working concentration of 100 nM. The MitoTracker™ Green FM solution was added to the wells, and cells were incubated for 15 min in a 37°C CO_2_ incubator. After staining, the MitoTracker™ Green solution was removed, and cells were washed with PBS to eliminate unbound dye.

Optical density (OD) readings were taken using a plate reader with excitation/emission wavelengths of 490/516 nm. Unstained cells served as background controls for normalization. Relative fluorescence units (RFUs) were calculated by subtracting the background fluorescence of control wells from that of treated wells.

### 2.15 Statistical analysis

Statistical analysis and scientific 2D graphing were performed using the commercially available statistics software package GraphPad Prism 10 (GraphPad Prism, CA, United States). The results from two groups were compared using a Student’s t-test. Comparisons between three or more groups in which there were two independent variables were analyzed using a two-way analysis of variance (ANOVA) with Bonferroni’s *post hoc*. Quantitative data is expressed as mean ± standard error of the mean (SEM). P < 0.05 was considered statistically significant for differences between the groups.

## 3 Results

### 3.1 TNFɑ and NRF2 exhibit opposing expression in HDP placentas

**FIGURE 1:**
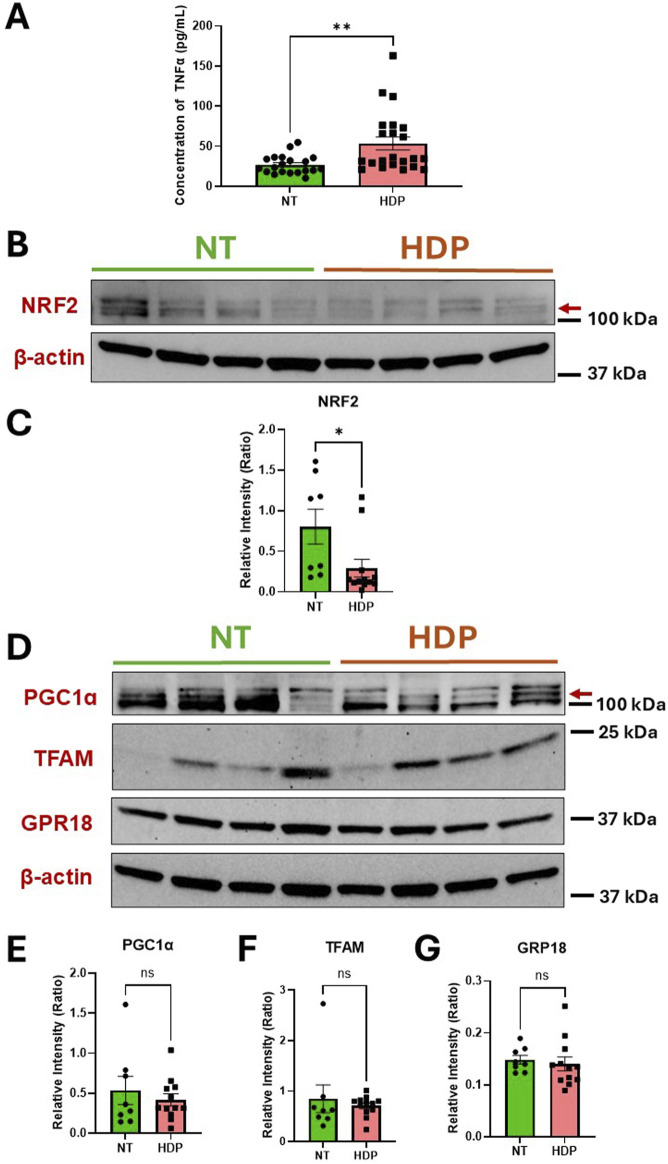
HDP human placental tissues exhibit increased TNFα concentrations but decreased NRF2 expression as compared to NT placental tissues. Protein expressions of TNFα and NRF2 were analyzed from human placental tissues in NT and those with HDP. **(A)** TNFα protein concentrations were determined by ELISA; *n* = 20–22 per group. **(B)** Immunoblot representative of NRF2. **(C)** Immunoblot analysis comparing NRF2 band intensities (red arrow) relative to β-actin; *n* = 8–12 per group. **(D)** Immunoblot representative for PGC1ɑ, TFAM, and GPR18. **(E,F and G)** Immunoblot analysis comparing PGC1ɑ, TFAM and GPR18 band intensities to β-actin; *n* = 8–12 per group; *p < 0.05 and **p < 0.01 compared to NT; Student’s t-test.

In this study we first evaluated the protein levels of TNFɑ and NRF2 in the human placental tissue of normotensive (NT) and HDP. Our observation revealed significantly higher TNFα concentrations in placental tissue from HDP subjects compared to NT subjects (Figure 1A). To resolve inconsistencies in NRF2 protein size reported in the literature (Lau et al., 2013), we treated placental trophoblasts with a proteasome inhibitor, MG-132 to increase NRF2 protein levels (Supplementary Figure S1). This allowed us to detect the correct NRF2 protein (∼100–110 kDa) in HDP placental tissues. Subsequently, our immunoblots revealed decreased NRF2 protein expression in HDP placentas compared to NT (Figures 1B,C). Since NRF2 can interact with PGC1ɑ and TFAM, we examined the protein expression of these transcription factors along with GPR18, which is a receptor for RvD2 (Figures 1D–G). We do not observe any changes in the levels of PGC1ɑ, TFAM and GPR18 in the placenta obtained from NT and HDP (Figures 1D–G) Regardless, these findings suggest potential crosstalk between TNFɑ and the NRF2 antioxidant response, highlighting NRF2 as a promising therapeutic target. Increasing NRF2 expression may mimic the role of antioxidants, emphasizing its potential in managing oxidative stress in placental tissues.

### 3.2 Effect of TNFα and RvD2 on the NRF2 signaling pathway in JEG-3 trophoblast cells

One of the most commonly observed pro-inflammatory cytokine in the placenta during HDP is TNFα (Benyo et al., 2001). Therefore, we tested TNFα as an inducer of oxidative stress in trophoblasts and explored the capacity of RvD2 to modulate TNFα-associated changes *in vitro*. To assess the effects of TNFα and RvD2 on the NRF2 signaling pathway in trophoblasts, cells were pre-treated with 100 nM RvD2 for 16 h, followed by exposure to 100 ng/mL TNF-α for 5 or 10 h (designated as 16 + 5 or 16 + 10), resulting in total incubation times of 21 or 26 h.

**FIGURE 2:**
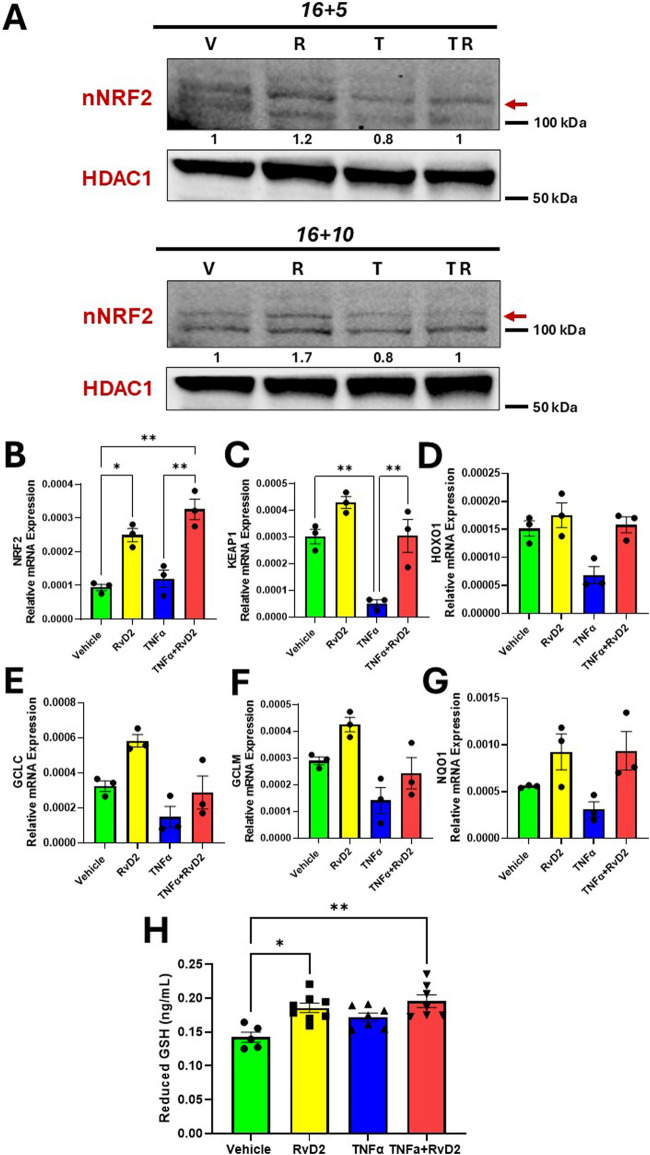
RvD2 upregulates the NRF2 signaling cascade in TNFɑ-induced JEG-3 cells. For cotreatment of TNFɑ + RvD2 (TR) groups, cells were pretreated with RvD2 for 16 h, followed by TNFɑ treatment for an additional 5 or 10 h (16 + 5 h or 16 + 10 h), resulting in total treatment durations of 21 and 26 h, respectively. For vehicle (V) or RvD2 (R) treatments, cells were treated for a total of 21 or 26 h. Cells treated with TNFɑ (T) were exposed for either 5 or 10 h. **(A)** Immunoblot analysis of NRF2 in 16 + 5 h and 16 + 10 h treatment strategies. The values below the immunoblot represent band intensity ratio of nNRF2/HDAC1. The same blot was used in Figure 7. **(B–G)** Relative mRNA expression of 16 + 5 h treatment strategy of kelch-like ECH-associated protein 1 (KEAP1), hemoxygenase 1 (HOXO1), glutamate-cysteine ligase catalytic subunit (GCLC), glutamate-cysteine ligase modifier subunit (GCLM), NADPH quinone oxidoreductase 1 (NQO1) in trophoblasts; *n = 3* per group. **(H)** Reduced glutathione was measured with the pretreatment of RvD2 (100 nM) for 16 h followed by a 1 h treatment of TNFɑ (100 ng/mL); *n =* 5–7 per group. Data presented as mean ± SEM; *p < 0.05 and **p < 0.01 compared against each treatment.

Figures 2A,B indicate that nuclear (n) NRF2 protein and mRNA expression were upregulated in the RvD2 treated cells and TNFɑ + RvD2 treated cells, indicating that RvD2 can activate NRF2. KEAP1 is normally a repressor of NRF2. Thus, we examined KEAP1 mRNA expression levels and observed an increase between the TNFɑ and TNFɑ + RvD2 treated cells. (Figure 2C). NRF2 activation is known to enhance the expression of antioxidant enzyme genes and promote GSH production. Therefore, we tested how RvD2 and TNFα differentially affected these genes at the transcription level. Among the Vehicle *versus* RvD2, Vehicle *versus* TNFα, and TNFα *versus* TNFα + RvD2 treated cells, we observed a non-significant difference but a trend in the mRNA expression of key enzymes involved in antioxidant defense, hemoxygenase 1, glutamate-cysteine ligase modifier subunit, and glutamate-cysteine ligase catalytic subunit, which are involved in GSH production. (Figures 2D–F). We detected a pattern in NADPH quinone dehydrogenase 1, an enzyme that helps to detoxify ROS, but there was no significant difference between groups (Figure 2G). Additionally, GSH concentrations were significantly elevated in the RvD2 treated cells compared to the Vehicle group. However, TNFɑ treated cells did not significantly alter the GSH concentrations compared to the Vehicle group (Figure 2H). These data suggest that RvD2 may target the NRF2 pathway, which can potentially protect cells from TNFα-induced oxidative stress.

### 3.3 RvD2 increases nuclear NRF2 localization expression levels in JEG-3 cells with and without TNFɑ

**FIGURE 3:**
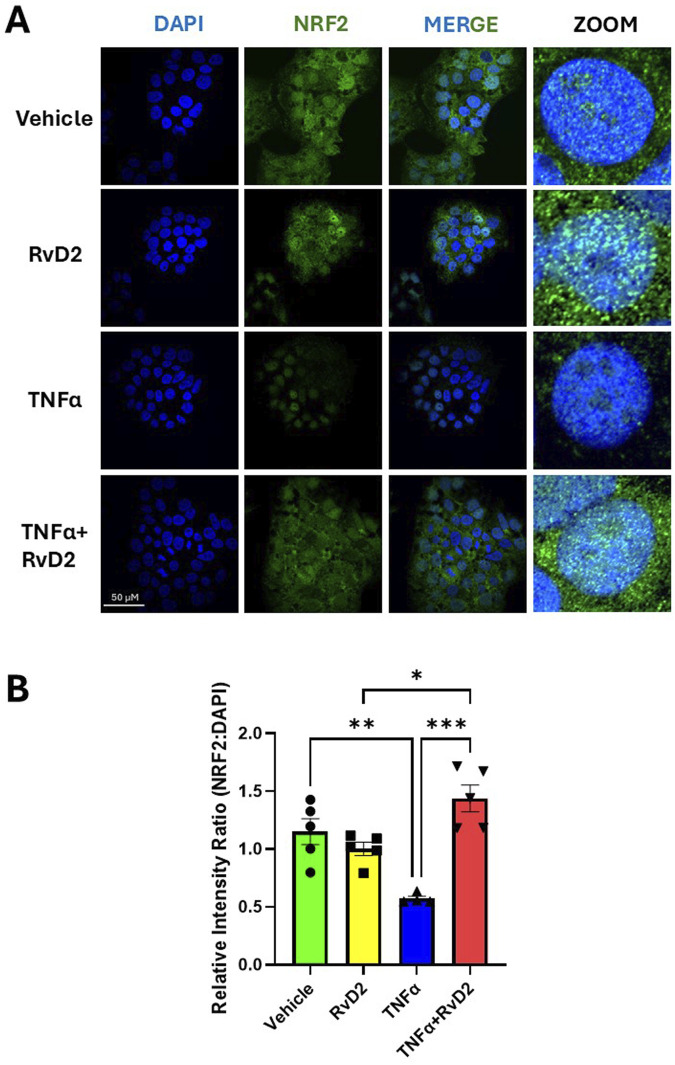
RvD2 increases NRF2 expression in the presence of TNFα after 24 h. JEG-3 cells were treated with 100 nM RvD2, 50 ng/mL TNFα, or a combination of RvD2 + TNFα for 24 h. **(A)** Immunofluorescence images: blue represents DAPI (nuclei), and green represents NRF2. Teal areas within the DAPI-stained regions in the merged and zoomed-in images indicate increased nuclear colocalization of NRF2 with cotreatment of TNFα and RvD2. **(B)** Quantification of relative intensity of NRF2 expression from images converted to binary format, compared to DAPI. At least 100 cells per treatment condition were analyzed for the relative intensity ratio of NRF2:DAPI. Data are presented as mean ± SEM; n = 4–5 per group; *p < 0.05, **p < 0.01, ***p < 0.001, compared to each treatment.

To assess whether RvD2 or TNFα can activate nuclear translocation of NRF2, we performed immunofluorescence imaging in trophoblasts. Trophoblasts were treated with 100 nM RvD2, 50 ng/mL of TNFɑ, or a cotreatment of TNFɑ + RvD2 for a total of 24 h. We observed an increased overlay of NRF2 and nuclei (DAPI) in trophoblasts following treatment with RvD2 compared to the Vehicle after 24 h (Figure 3A). Treatment with TNFα in trophoblasts resulted in a significant reduction in NRF2 nuclear translocation after 24 h compared to Vehicle (Figure 3A). However, this effect was mitigated when TNFα was cotreated with RvD2, as indicated by the increased teal signal in the merged image (Figure 3B). Although no significant difference was observed at 8 h, there is a noticeable downward trend in the TNFα group (Supplementary Figures S2A,B). These findings suggest that NRF2 translocated to the nucleus despite oxidative insult, with overall NRF2 expression upregulated in response to RvD2 treatment (Figure 3B). These results support the role of NRF2 nuclear translocation in facilitating binding to AREs.

### 3.4 RvD2 mitigates TNFα-induced mitochondrial ROS in JEG-3 trophoblast cells

**FIGURE 4:**
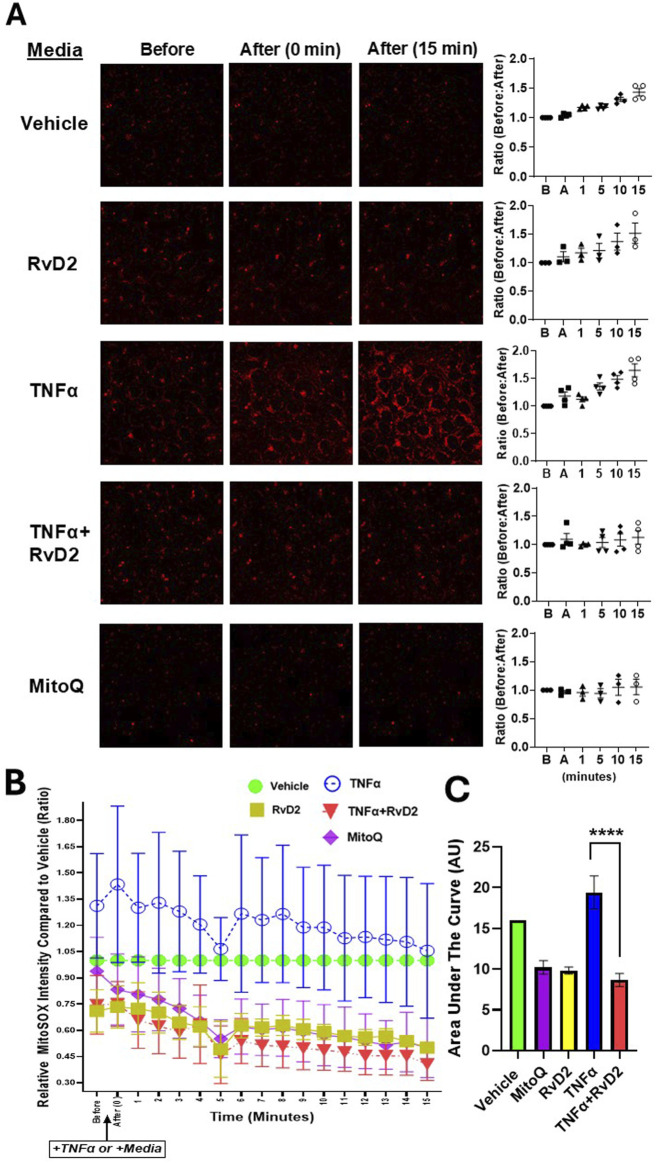
RvD2 mitigates TNFα-induced mitochondrial ROS in trophoblast cells. Relative ROS intensity was measured using MitoSOX fluorescent dye. **(A)** Representative live-cell confocal microscopy images of JEG-3 cells pretreated for 16 h with 100 nM RvD2, 10 µM Mitoquinone mesylate (MitoQ) as a positive control, or Vehicle. After pretreatment, cells were exposed to 100 ng/mL TNFα “*+*T*NFα”* or medium “*+Medium*” for 15 min during live-cell confocal imaging. Images were taken immediately after treatment and 15 min post-treatment following the partial medium replacement with either warm TNFα or medium. The intensity ratios of ROS were calculated at baseline/before “B” and post-treatment/after “A” at 1, 5, 10, and 15-min intervals, with a baseline ratio value set to 1. **(B)** A line graph showing ROS intensity over time for each treatment group, compared to the Vehicle controls from the same day, and comparing the before and after images for each group. **(C)** The accompanying bar chart illustrates the area under the curve (AUC) in arbitrary units (AU), where Vehicle is set to one. Data are presented as mean ± SEM; *n* = 3–4 per group; ****p < 0.0001, compared against MitoQ, TNFɑ, RvD2, and TNFɑ + RvD2.

To confirm whether RvD2 promotes redox homeostasis by counteracting ROS, we examined its ability to functionally protect cells from ROS-induced damage. Trophoblasts were pre-treated with 100 nM RvD2 for 16 h, followed by exposure to 100 ng/mL TNF-α for 15 min during live-cell confocal imaging. Mitoquinone mesylate (MitoQ, 10 µM), a known ROS scavenger, was used as a positive control with a 16-h pre-treatment strategy. Pretreatment with RvD2 for 16 h effectively prevented mitochondrial superoxide formation, with effects similar to those observed with the ROS scavenger, MitoQ (Figure 4A). MitoSOX relative fluorescence intensity was dramatically increased following TNFα (100 ng/mL) exposure, indicating the activation of superoxide generation in the mitochondria (Figure 4A). A 16-h pretreatment of RvD2 followed by TNFα dramatically decreased mitoSOX relative fluorescence intensity over time (Figure 4B). The area under the curve for the intensity over time graph was similar between MitoQ, RvD2-treated cells, and the combination of TNFα + RvD2 treated cells (Figure 3C). It is important to note that, since groups were compared to the Vehicle on the same day of imaging, all the data reported as fold change and were normalized to the Vehicle treated cells (Figures 4B,C). Comparing the Before:After image intensity ratio based on the respective image highlights the importance of normalizing data to account for the Vehicle on that day (Supplementary Figure S3). Overall, this data suggests that RvD2 mitigates TNFα-induced increases in mitochondrial superoxide generation.

### 3.5 RvD2 reduces TNFα-induced oxygen consumption rates without changing its electron transport chain complexes

**FIGURE 5:**
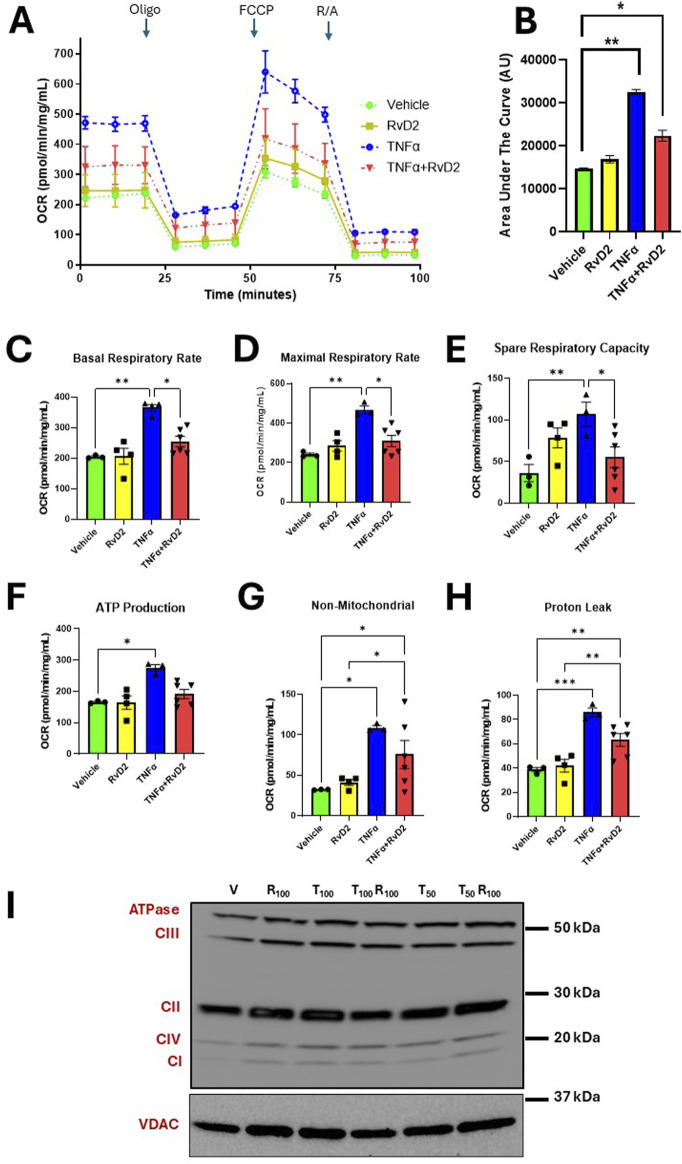
Pretreatment of RvD2 reduces TNFα-induced oxygen consumption rates (OCR) without altering electron transport chain (ETC) proteins. Bioenergetic Seahorse™ analysis of JEG-3 cells pretreated for 16 h with Vehicle or 100 nM RvD2, followed by a 5-h treatment of 100 ng/mL TNFα. **(A)** OCR levels were measured during injections of Oligomycin (Oligo; 1 μM), Carbonyl cyanide-p-trifluoromethoxyphenylhydrazone (FCCP; 1 μM), and Rotenone/Antimycin A (R/A; 0.5 μM). **(B)** Area under the curve (AUC) in arbitrary units (AU). **(C–H)** Comparison of OCR values for basal, maximal, and spare respiratory capacity, proton leak, ATP production, and non-mitochondrial respiration between groups, *n =* 3–6 per group. **(I)** Immunoblot representative images showing mitochondrial protein fractions for total ETC complex proteins from JEG-3 cells pretreated with Vehicle (V) or 100 nM RvD2 (R_100_), followed by a 5-h treatment with 50-100 ng/mL TNFα (T_50_ or T_100_). Data are presented as mean ± SEM; *p < 0.05, **p < 0.01, and ***p < 0.001.

We next measured oxygen consumption rates (OCR) to investigate whether RvD2 has a protective effect on mitochondrial efficiency and energy production in trophoblasts. Cells were pre-treated with 100 nM RvD2 for 16 h, followed by exposure to 100 ng/mL TNF-α for 5 h. OCR changes were observed with the respective, ETC modulators (Figure 5A). Comparing the treatment groups, a significant difference was observed between Vehicle and RvD2. Additionally, significant differences were found when comparing Vehicle to TNFɑ, as well as between Vehicle and TNFɑ + RvD2. We also measured OCR graph’s area under the curve (AUC), which showed increased OCR area under the curve with TNFα and decreased AUC with co-treatment of RvD2 + TNFα (Figure 5B). RvD2 treated cells did not alter bioenergetic parameters compared to the Vehicle group. However, TNFɑ markedly elevated bioenergetic parameters, including basal respiration, maximal respiration, spare respiratory capacity, ATP production, non-mitochondrial respiration, and proton leak, relative to Vehicle-treated cells. A 16-h pretreatment with RvD2, followed by a 5-h TNFɑ insult, significantly attenuated TNFɑ-induced increases in OCR, particularly in basal respiratory rate, maximal respiratory rate, and spare respiratory capacity. However, RvD2 pretreatment alone did not alter TNFɑ-induced increases in ATP production, non-mitochondrial respiration, or proton leak (Figures 5C–H). We next measured specific electron transport chain (ETC) complex protein levels such as Complex I, II, III, IV, and ATPase/V, (Figure 5I). These, ETC proteins remain unchanged. This suggests that the increased energy demands observed in TNFɑ-treated cells are likely driven by altered activity or interactions between ETC complexes, rather than changes in mitochondrial protein levels. Our data suggest that RvD2 pretreatment likely preserved the integrity of the inner mitochondrial membrane, reducing proton leak, and stabilizing, ETC function.

### 3.6 RvD2 enhances mitochondrial function in a TNFɑ stimulated environment

**FIGURE 6:**
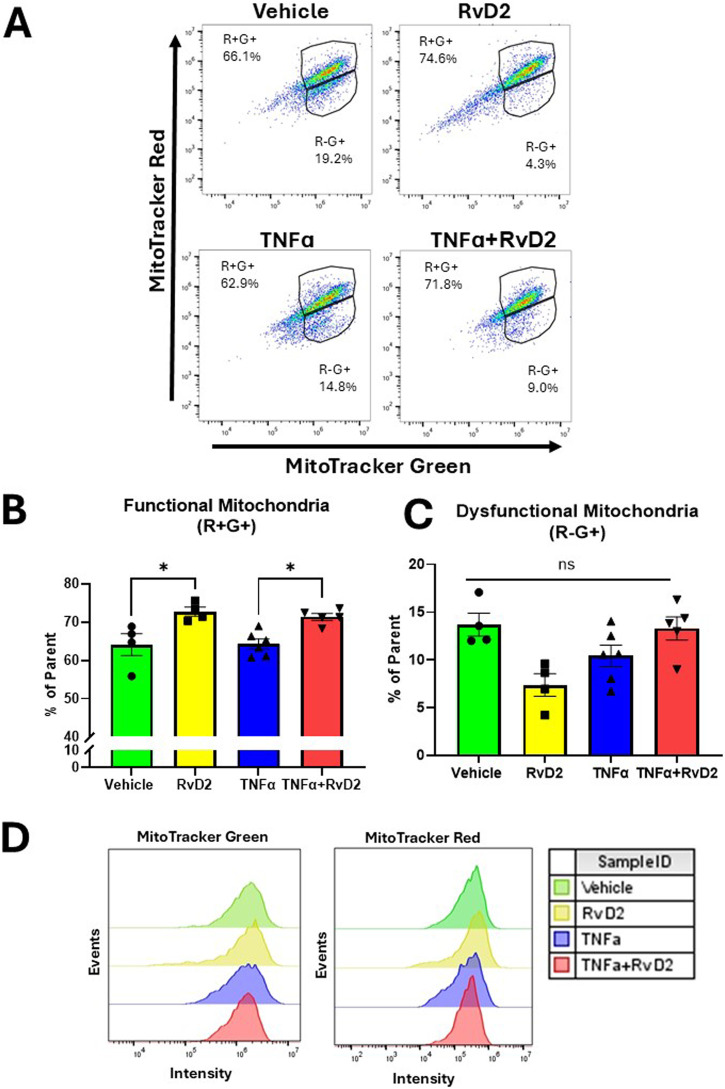
RvD2 improves mitochondrial function in a TNFɑ-induced environment in trophoblasts. JEG-3 cells were pretreated with 100 nM of RvD2 for 16 h, followed by a 1-hour exposure to 100 ng/mL of TNFɑ. **(A)** Representative dot plot analysis of cell populations stained with MitoTracker™ Green and MitoTracker™ Red CMXRos using flow cytometry. **(B)** Functional mitochondria were identified as MitoTracker™ Red ^high (+)^ and MitoTracker™ Green ^high (+)^, denoted as “R+G+”. **(C)** Cells with dysfunctional mitochondria were classified as MitoTracker™ Red ^low (−)^ and MitoTracker™ Green ^high (+)^, denoted as “R−G+”. **(D)** Representative flow cytometry median peak intensity graphs (events vs. MitoTracker™ Green fluorescence intensity and events vs. MitoTracker™ Red fluorescence intensity). Data presented as mean ± SEM; n = *4−5* per group; *p < 0.05.

To assess mitochondrial activity during RvD2 and TNF-α treatments, we performed flow cytometry in trophoblasts. Cells were pre-treated with 100 nM RvD2 for 16 h, followed by exposure to 100 ng/mL TNF-α for 1 h. The dot plots in Figure 6A illustrate populations of trophoblasts, gated based on mitochondrial mass and mitochondrial membrane potential. Percentages for each treatment condition were calculated relative to the parent population of live, singlet populations per the gating strategy (Supplementary Figure S4).

In the R+G subset population, a significant increase was observed in the RvD2 treated cells compared to Vehicle. In the same subset population, no significant difference was detected between the Vehicle and TNFɑ treated cells. Importantly, pretreatment with RvD2 followed by TNF-α exposure significantly enhanced R+G+ levels (Figure 6B). We did not find a significant difference between treatment groups in the R-G+ subset population (Figure 6C). An assessment of event intensities for MitoTracker Green and Red was included (Figure 6D). These data suggest that pretreatment with RvD2 can enhance mitochondrial mass and membrane potential in a TNFɑ treated environment, indicating a modulatory effect of RvD2 on mitochondrial function in the presence of TNFɑ.

### 3.7 RvD2 enhances mitochondrial activity and trophoblast cell migration in a TNFɑ-stimulated environment

Given that PGC1ɑ interacts with NRF2/NRF1 to enhance the expression of genes critical for mitochondrial biogenesis. A downstream target of the NRF2/PGC1α pathway is TFAM, which plays an essential role in mtDNA replication, transcription, and maintenance, thereby supporting proper mitochondrial function. NRF2 and PGC1α play a crucial role in cellular energy metabolism, which is integral to migration. Cell migration is an ATP-driven process, with PGC1α promoting mitochondrial biogenesis to ensure sufficient ATP production for supporting cytoskeletal rearrangements and the activation of signaling pathways required for cell movement (Cho et al., 2022; Valcarcel-Jimenez et al., 2019; Grieco et al., 2022).

To assess the effects of TNFα and RvD2 on PGC1ɑ and TFAM in trophoblasts, cells were pre-treated with 100 nM RvD2 for 16 h, followed by exposure to 100 ng/mL TNF-α for 5 or 10 h (designated as 16 + 5 or 16 + 10), resulting in total incubation times of 21 or 26 h. After the incubation periods, the cells were collected, and nuclear proteins were extracted.

**FIGURE 7:**
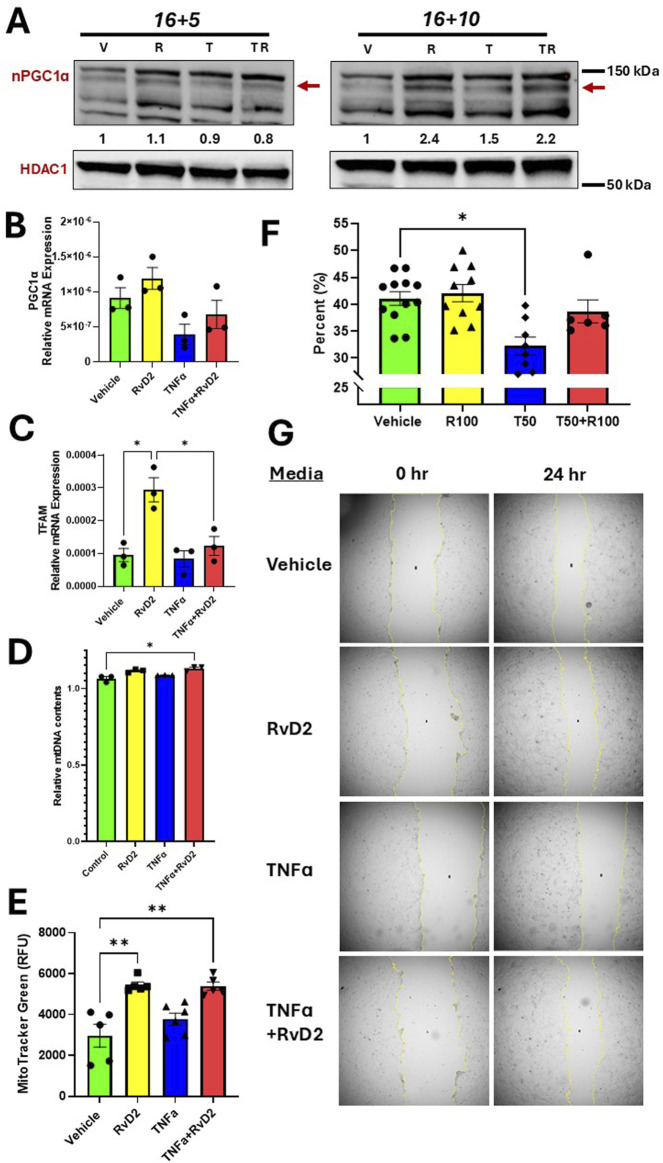
RvD2 stimulates mitochondrial biogenesis, increases mitochondrial content and improves migration. For cotreatment of TNFɑ + RvD2 (TR) groups, cells were pretreated with RvD2 for 16 h, followed by TNFɑ treatment for an additional 5 or 10 h (16 + 5 h or 16 + 10 h), resulting in total treatment durations of 21 and 26 h, respectively. For vehicle (V) or RvD2 (R) treatments, cells were treated for a total of 21 or 26 h. Cells treated with TNFɑ (T) were exposed for either 5 or 10 h. **(A)** Immunoblot representative of treatments. The values below the immunoblot represent band intensity ratio of PGC1ɑ/HDAC1. The same blot was used in Figure 2. **(B)** RT-qPCR results for 16 + 5 h treatment strategy of PGC1α, **(C)** TFAM, and **(D)** Relative mitochondrial DNA (mtDNA) content was compared by taking the ratio of nuclear DNA to mtDNA relative mRNA expression; *n =* 3 per group. **(E)** MitoTracker™ Green relative fluorescence units (RFU) were measured using the 16 + 5 h treatment strategy. For the migration assay, cells were treated for 24 h with Vehicle, 100 nM of RvD2, 50 ng/mL TNFɑ, and RvD2 + TNFɑ; *n =* 6–*1*2 per group. **(G)** Migration bar chart (percentages, %) of various groups; *n* = 6–12. **(F)** Representative images of the scratch assay. Data are presented as mean ± SEM; *p < 0.05 and **p < 0.01.

PGC1α protein expression is increased in the 16 + 10-h pretreatment strategy for Vehicle *versus* RvD2 treated cells and the TNFɑ *versus* TNFɑ + RvD2 treated cells (Figure 7A). mRNA expression of PGC1ɑ was not statistically significant when comparing Vehicle to RvD2, Vehicle to TNFɑ, and TNFɑ to TNFɑ + RvD2 (Figure 7B). However, the downstream target TFAM showed a significant difference in mRNA expression between Vehicle and RvD2 treated cells, but not between Vehicle and TNFɑ or between TNFɑ and TNFɑ + RvD2-treated cells (Figure 7C). Next, we examined if RvD2 would alter mtDNA levels. We observed no significant differences between the Vehicle and RvD2 groups, the Vehicle and TNFɑ groups, or the TNFɑ and TNFɑ + RvD2 groups (Figure 7D). We explored how RvD2 would alter mitochondrial mass by using MitoTracker Green and discovered a significant difference between Vehicle and RvD2 but not between Vehicle and TNFɑ, or TNFɑ and TNFɑ + RvD2 treated cells (Figure 7E). Finally, we investigated the role of the effects of RvD2 and TNFα on trophoblast migration. Our data revealed that 50 ng/mL of TNFα significantly inhibited cell migration compared to the Vehicle treated cells (Figures 7F,G). Taken together, these data suggest that RvD2 may be increasing mitochondrial biogenesis, replication, and mitochondrial mass, while TNFɑ inhibits migration. Pretreatment with RvD2 may slightly, but not significantly, alter these functions.

## 4 Discussion

Inflammation-related oxidative stress in placenta and maternal decidua are key contributors to the pathogenesis of HDP (Hahka et al., 2024; Sultana et al., 2023). TNFɑ is a known inducer of oxidative stress and mitochondrial dysfunction (Marin et al., 2020; Smith et al., 2021; Dos Passos Junior et al., 2021; Wang and Walsh, 1996; Lyu et al., 2023). While anti-inflammatory and antioxidant strategies show promise, nutrient-based approaches are hindered by limited understanding of their molecular targets. Omega-3 fatty acids-derived SPMs such as RvD2 are known to reduce inflammation and promote tissue healing (Ulu et al., 2019; Mas et al., 2012). This study fills a gap in knowledge regarding the protective role of RvD2’s ability to protect against oxidative stress and mitochondrial dysfunction in placental trophoblasts.

In the present study, our findings indicated that RvD2 is a protective nutrient-derived metabolite which functioned as a positive regulator of endogenous antioxidants, reduced ROS, and enhanced mitochondrial function. The main findings of the study were as follows: 1) NRF2 was downregulated in human placental tissue, while TNFɑ concentrations were increased in HDP; 2) pretreatment of RvD2 activated NRF2 signaling in trophoblast; 3) RvD2 mitigated TNFα-induced ROS in trophoblasts; and 4) RvD2 attenuated TNFɑ-induced increased mitochondrial OCR. The proposed signaling mechanisms of RvD2 and TNFα are illustrated in a schematic diagram in graphical abstract.

### 4.1 NRF2 is downregulated in human placental tissue while TNFɑ concentrations are increased in HDP

Consistent with previous studies, our findings confirm that elevated TNFα concentrations are present in HDP placentas (Wang and Walsh, 1996; Wardhani, 2022; Kalantar et al., 2013). TNFɑ’s production is typically triggered by stressors such as high blood pressure (Chen et al., 2022). TNFɑ can originate from various sources, including macrophages, monocytes, and other immune cells that infiltrate into the placenta (Jang et al., 2021). This presents a challenge, as TNFα plays a crucial regulatory role in placentation, so production must be modulated in pregnancy but not completely suppressed. TNFα interacts with its receptors, TNFR1 and TNFR2, which mediate various cellular processes including altered calcium handling, matrix metalloproteinase activation, apoptosis, necroptosis, and the release of additional proinflammatory cytokines (C-C motif ligand cytokines or CCLs) (Agostini et al., 1995).

Of note, HDP is accompanied with elevated levels of other proinflammatory cytokines, such as IL-6 and IL-17, as well as reduced anti-inflammatory cytokines, including IL-10 and IL-4 (Ulu et al., 2019; Maseliene et al., 2023; Fakhr et al., 2022). However, given that TNFα concentrations tend to progressively increase with the severity of HDP during pregnancy (Wang et al., 2018), we chose to focus on TNFα as the primary inducer of insult. TNFα treatments have been verified to induce preeclamptic-like phenotypes in placental villi (Fakhr et al., 2022). TNFɑ concentration used in the present study is of pathophysiological relevance, as mean systemic circulating levels are 44.52 ng/mL in normal pregnancies, 63.33 ng/mL in mothers with PE, and 117.18 ng/mL in severe PE with sepsis (Wardhani, 2022).

In the present study, we established that NRF2 expression is significantly reduced in HDP placentas compared to normotensive placentae, consistent with findings from other studies (Chigusa et al., 2012; Zakeri et al., 2024). Decreased NRF2 expression is likely linked to reduced activity, impaired antioxidant responses, elevated ROS, and placental mitochondria dysfunction, which are hallmark features of HDP. Notably, TNFα has been shown to interact with NRF2, suppressing its activity—a relationship similarly observed in cardiomyocytes (Shanmugam et al., 2016). Further studies are required to elucidate whether decreased NRF2 during term placenta from individuals with HDP were also observed in the first and second trimester of pregnancy.

### 4.2 TNFɑ decreases NRF2 and increases ROS and OCR in trophoblasts

In our study, we modeled HDP *in vitro* by exposing trophoblasts to TNFα to induce oxidative stress. With these treatments, we demonstrated that TNFα increases mitochondrial ROS production, enhances OCR in trophoblast cells, and decrease cell migration. These effects may result from TNFα′s ability to increase ETC activity, elevating oxygen consumption as the terminal electron acceptor. While ROS are known as signaling molecules, their production inherently contributes to an increased OCR and increased proton leak (Averill-Bates, 2024). Similarly, in the present study, treatment of TNFɑ did increase proton leak in trophoblasts. Alternatively, this effect could be explained by a metabolic shift from OXPHOS to glycolysis to meet the rapid energy demands of inflammation, enhancing OCR to support ATP production during oxidative stress—an observation similarly reported in microglial and hepatic cells (Cheng et al., 2021; Kastl et al., 2014). However, further studies are required to discuss how RvD2 mitigates this metabolic shift from glycolysis back to OXPHOS.

### 4.3 RvD2 upregulated NRF2, enhanced glutathione levels, and mitochondrial function in trophoblasts

Activation of NRF2 protects against oxidative stress (Bellezza et al., 2018). When comparing RvD2 to the Vehicle treated cells, we observed that trophoblasts exhibited upregulation of NRF2 protein and gene expression, KEAP1 mRNA expression, enhanced GSH production, improved mitochondrial function, increased PGC1ɑ protein expression, and elevated TFAM mRNA expression.

The parallel increase in NRF2 and KEAP1 was unexpected but may result from the dissociation of NRF2 and KEAP1 (Dodson et al., 2019; Lin et al., 2023; Bellezza et al., 2018; Ulasov et al., 2022). Under normal conditions, KEAP1 targets NRF2 for degradation (Lin et al., 2023; Bellezza et al., 2018; Ulasov et al., 2022; Shen et al., 2023; Harvey et al., 2009). ROS and electrophilic agents (aldehydes or hydroxyl radicals) are thought to disrupt the KEAP1-NRF2 interaction, allowing NRF2 to accumulate and activate antioxidant responses, with KEAP1 upregulated to prevent excessive activity (Ulasov et al., 2022). However, RvD2 is not an electrophilic agent, it works by activating GPR18, which had been discovered to induce intracellular cAMP, phosphoinositide 3-kinases-protein kinase B, and mitogen-activated protein kinases/extracellular signal-regulated kinases, while also influencing intracellular Ca^2+^ levels (Zhao et al., 2023). Our findings address a critical gap in demonstrating RvD2’s true ability to act as a positive regulator of endogenous antioxidants.

### 4.4 Pretreatment of RvD2 upregulated NRF2, mitigated ROS, and reestablished OCR levels in TNFɑ treated environment in trophoblasts

Mitochondrial OCR and the generation of ROS are crucial indicators of mitochondrial health. Both elevated and decreased OCR can negatively impact cellular function and overall health (Maltepe and Saugstad, 2009). Severe deviations in OCR, either an increase or a decrease, often lead to the overproduction of ROS, resulting in oxidative stress and damage to cellular structures (Maltepe and Saugstad, 2009). Therefore, maintaining a balance in OCR is essential for cellular health. Interestingly, our findings demonstrated that pretreatment with RvD2 mitigated TNFα-induced increased OCR in trophoblasts. Furthermore, RvD2 effectively blocked the production of ROS caused by TNFα insults in trophoblasts, highlighting its potential protective role against oxidative stress in these cells.

Mitochondrial membrane potential and mass are indicators to measure mitochondrial function (Monteiro et al., 2020). We discovered improvement in mitochondrial function when comparing TNFɑ treated cells to RvD2-pretreated TNFα-exposed trophoblasts. Functional mitochondria can signal mitochondrial biogenesis, prompting our investigation of the key biogenetic marker, PGC-1α, which is known to co-transcribe with NRF2 (Abu et al., 2023; Dillon et al., 2012). Literature has demonstrated that antioxidants, such as resveratrol can induce mitochondrial biogenesis (Csiszar et al., 2009). A previous study in muscle cells demonstrated that omega-3 fatty acids, eicosapentaenoic acid and docosahexaenoic acids, can modulate mitochondrial biogenesis (Lee et al., 2016). In this study we show that pretreated RvD2 + TNFɑ insulted cells increased the protein expression of NRF2 and PGC1ɑ compared to TNFɑ treated trophoblasts. Suggesting that NRF2 is the major player in regulating mitochondrial function. Compared to TNFɑ treatments, we also observed that RvD2-pretreated TNFα-exposed trophoblasts showed increased NRF2 mRNA and protein expression, accompanied by nuclear translocation of NRF2. This nuclear translocation suggests an impact on the ARE. However, further studies are necessary to differentiate the roles of NRF2 *versus* NRF1 and to examine the involvement of KEAP2.

Literature supports that the upregulation of PGC-1α and NRF2 correlates with improved cell migration (Cho et al., 2022; Zhang et al., 2019). Given the primary role of trophoblast cells in migration, we observed that a pretreatment of RvD2 did not significantly alter TNFɑ-induced insults although a trend was observed. However, in other cases, changes in PGC1α expression can promote cellular migration, suggesting a more nuanced role depending on the tissue type and environmental conditions (Zhu et al., 2009).

### 4.5 Limitations and future directions

These findings align with previous studies linking RvD2, TNFɑ-induced oxidative stress, and the NRF2–PGC1α–TFAM axis. They underscore RvD2’s potential as a novel therapeutic strategy against mitochondrial dysfunction, highlighting its role in regulating endogenous antioxidants in placental trophoblasts.

The observed reduction in NRF2 levels in HDP placental tissue may not solely result from TNFα. Term placental tissue homogenates encompass diverse cell types and hormones, and other factors such as fetal sex or environmental exposures (e.g., ultrafine particles) may also contribute to NRF2’s reduction (Behlen et al., 2022). We acknowledge that numerous cellular events and molecular mechanisms can influence mitochondrial biogenesis. However, exploring all related pathways, such as mitophagy, and others, is beyond the scope of this study.

Our findings suggest that RvD2 protects mitochondria from TNFɑ-induced insults; however, further research should investigate the specific mechanisms by which RvD2 exerts this protective effect. Such studies should include an exploration of the metabolic pathways involved to determine whether RvD2 contributes to the upregulation of β-oxidation or other metabolic routes. Although PGC1ɑ is linked to lipid metabolism and long-chain fatty acid oxidation, its precise role in the context of RvD2 requires further investigation (Cheng et al., 2018).

Additionally, it is important to examine whether RvD2 affects mitochondrial membrane lipid composition. Omega-3 fatty acids have also been shown to enrich the membrane phospholipids and impact lipid remodeling (Chang et al., 2018). However, further research is necessary to evaluate if RvD2 can impact phospholipid structures in the mitochondria or mitochondrial dynamics such as fission or fusion. In human airway cells, TNFɑ has been shown to increase mitochondrial fragmentation through the activation of dynamin-related protein 1, while reducing mitofusin 1 levels (Delmotte et al., 2021). It is plausible that RvD2 mitigates TNFɑ-induced mitochondrial damage by preventing fragmentation, thereby enhancing mitochondrial biogenesis and overall function.

### 4.6 Conclusion

In conclusion, our study addresses a critical research gap by demonstrating that RvD2 mitigates TNFα-induced oxidative stress while upregulating the NRF2 signaling pathway and improving mitochondrial function. The NRF2 pathway holds therapeutic potential for HDP, especially given that NRF2 is often downregulated and TNFα upregulated in human placental tissues. These findings suggest that targeting the NRF2 signaling cascade with RvD2 could provide a promising approach to counter the effects of TNFα in HDP, warranting further studies to develop RvD2-based treatments for TNFα-insulted placental trophoblasts.

## Data Availability

The original contributions presented in the study are included in the article/Supplementary Material, further inquiries can be directed to the corresponding authors.

## Abbreviations

ARE, Antioxidant response element; GCLC, glutamate-cysteine ligase catalytic subunit; GCLM, glutamate-cysteine ligase modifier subunit; GSH, Glutathione; GPR18, G-protein coupled receptor-18; HDP, Hypertensive disorders of pregnancy; HOXO1, hemoxygenase 1; KEAP1, kelch-like ECH-associated protein 1; KEAP2, kelch-like ECH-associated protein 2; mtDNA, Mitochondrial DNA; NRF1, Nuclear Factor Erythroid 2-Related Factor 1; NRF2, Nuclear Factor Erythroid 2-Related Factor 2; NQO1, NADPH quinone oxidoreductase 1; PGC1ɑ, Peroxisome Proliferator-Activated Receptor Gamma Coactivator 1 Alpha; PE, Preeclampsia; ROS, Reactive Oxygen Species; Rv, Resolvin; RvD2, Resolvin D2 Series; SPMs, Specialized pro-resolving lipid mediators; TFAM, Mitochondrial Transcription Factor A; TNFɑ, Tumor Necrosis Factor Alpha.
