# Supplementary material for: RvD2 mitigates TNFɑ-Induced mitochondrial reactive oxygen species through NRF2 signaling in placental trophoblasts
Source: Front Physiol. 2025 Apr 2;16:1547940. doi: 10.3389/fphys.2025.1547940 (PMC12000658; doi:10.3389/fphys.2025.1547940)
Supplement: Supplementary file 1 [file DataSheet2.pdf]

Figure 1B:
NRF2
Human Placenta

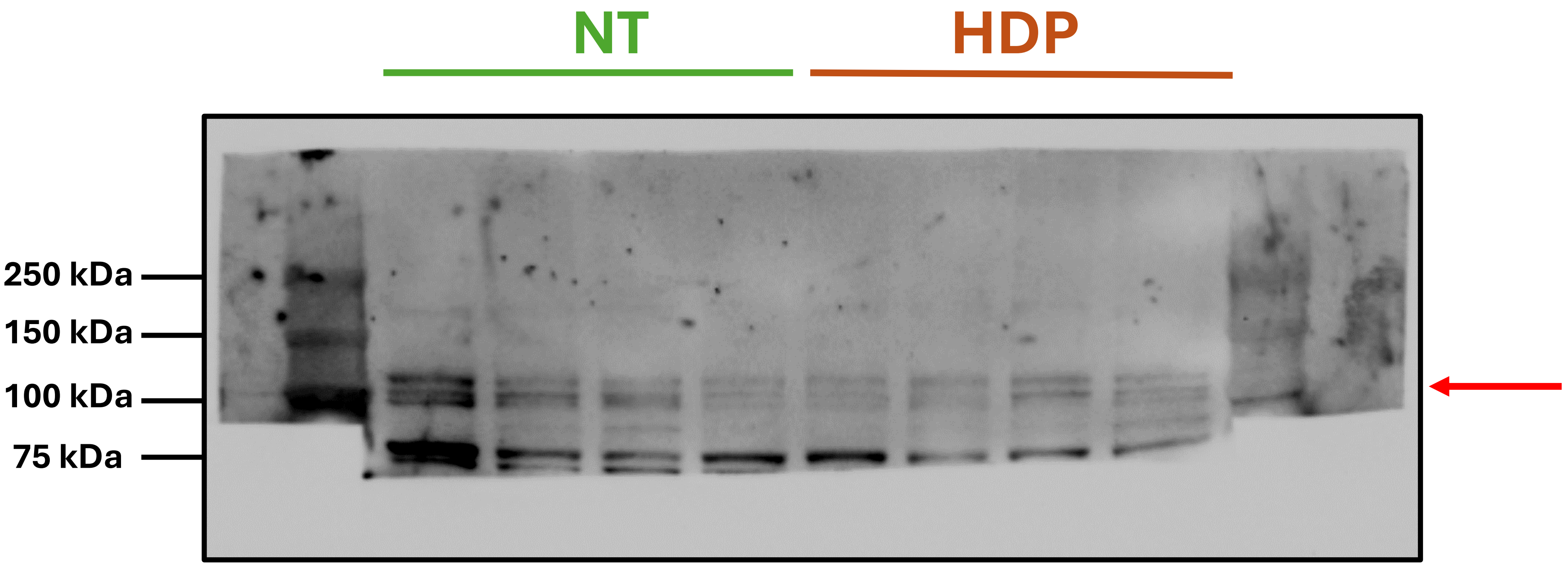

Figure 1B:
B-actin
Human Placenta

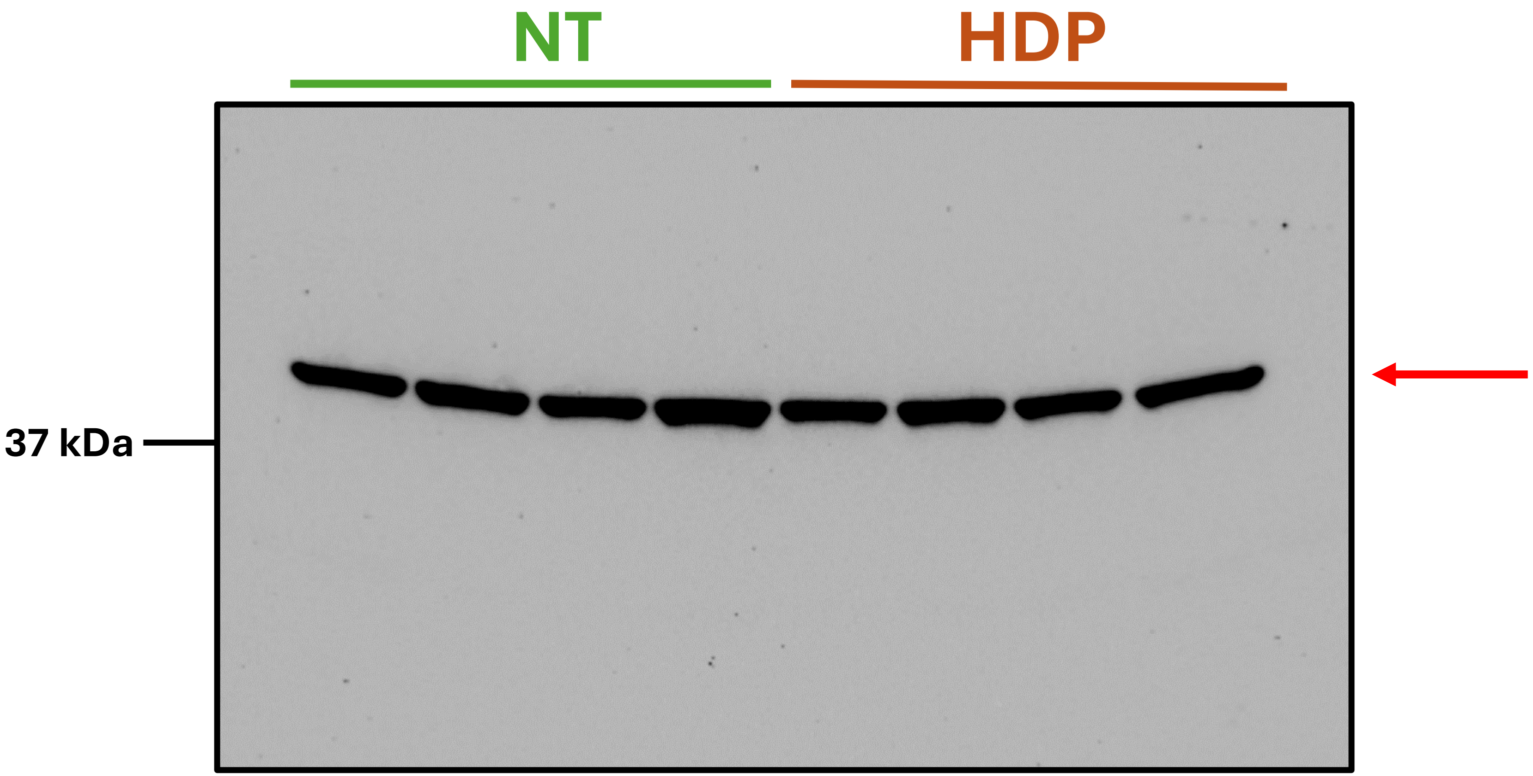

Figure 1D:
PGC1a
Human Placenta

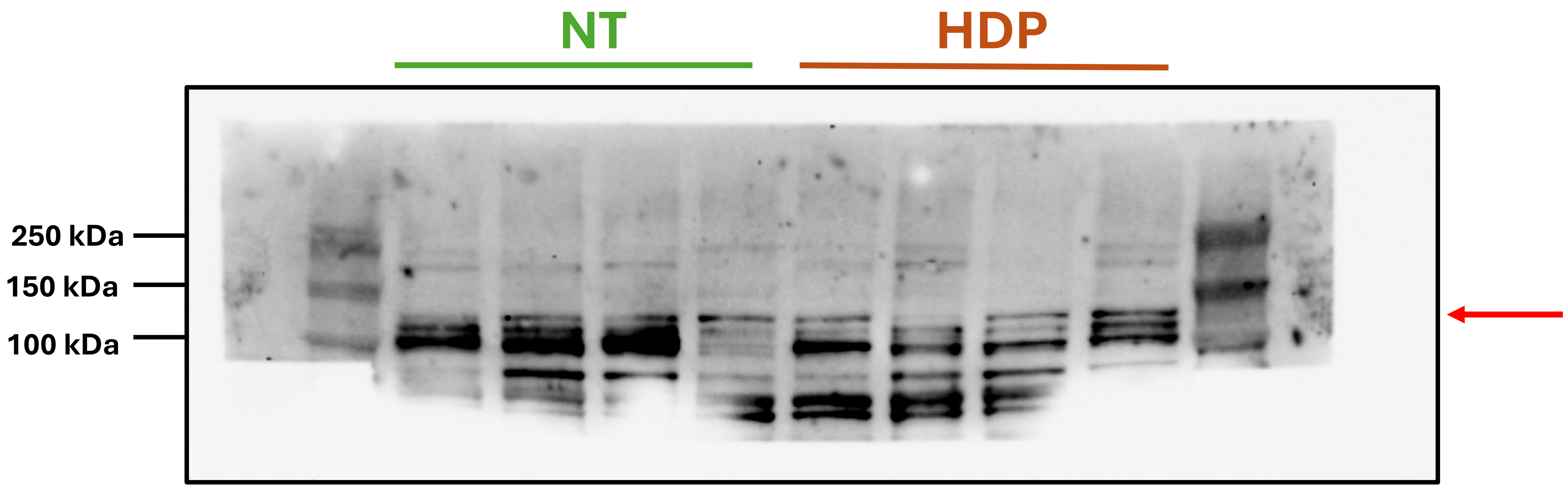

Figure 1D:
TFAM
Human Placenta

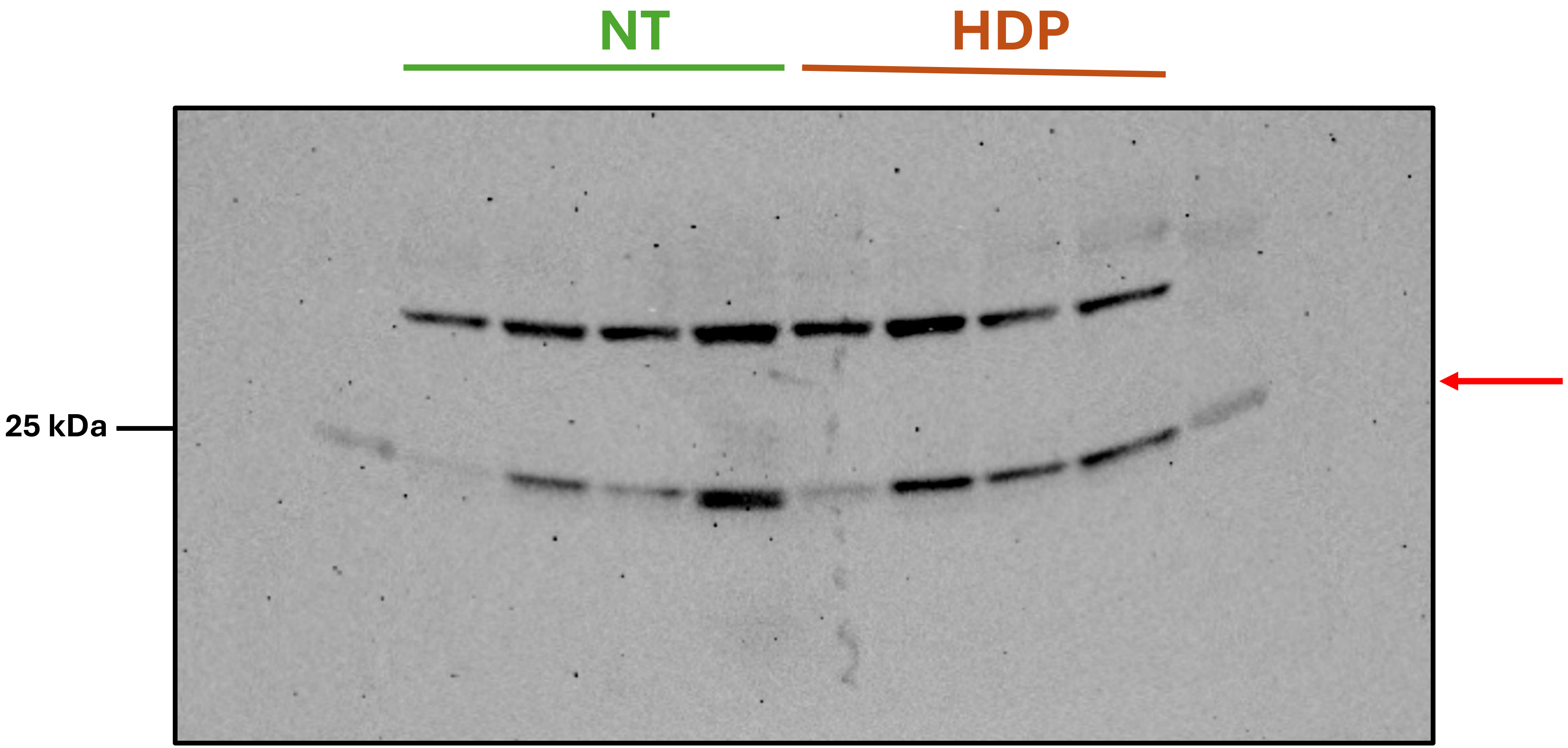

Figure 1D:
GRP18
Human Placenta

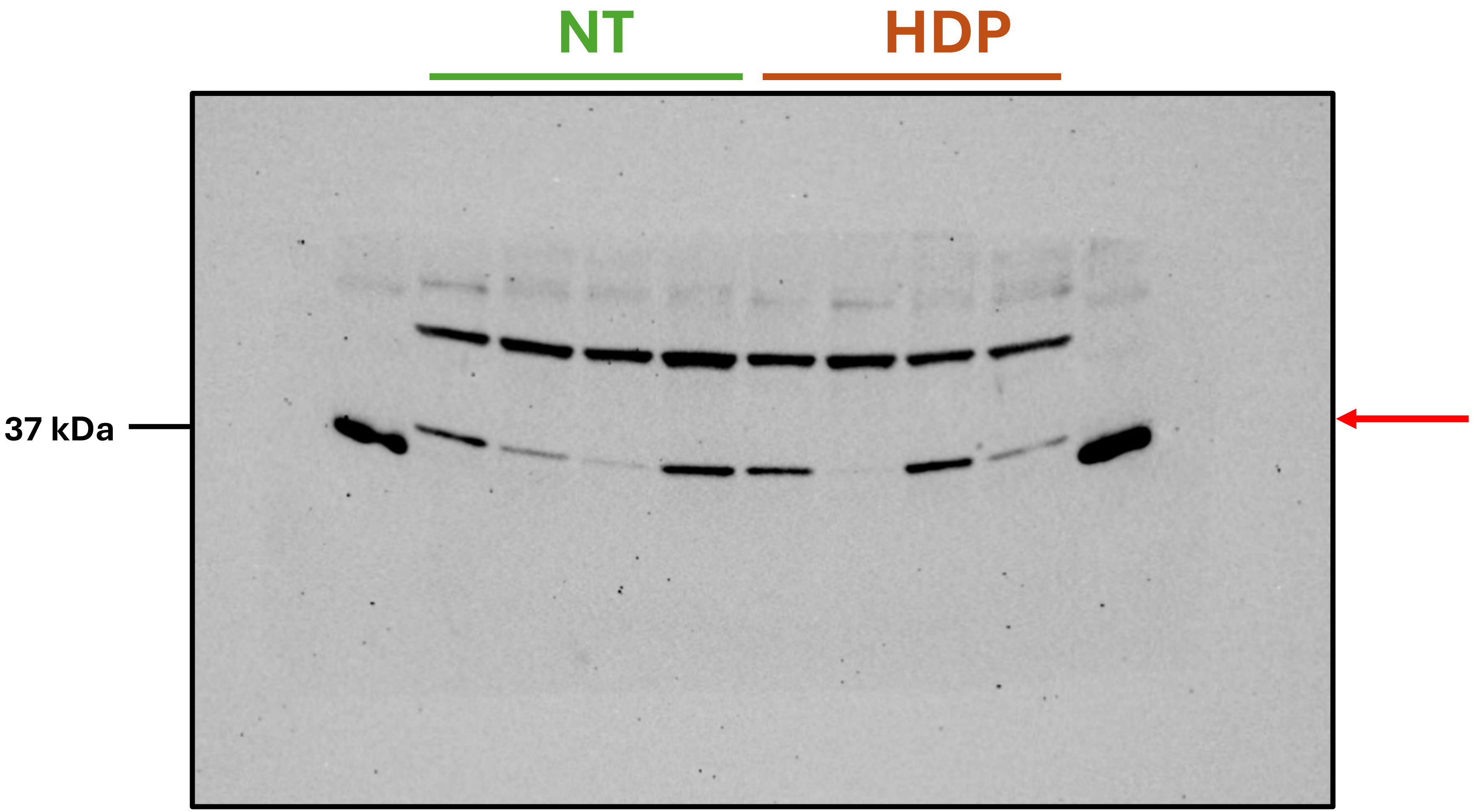

Figure 1D:
B-actin
Human Placenta

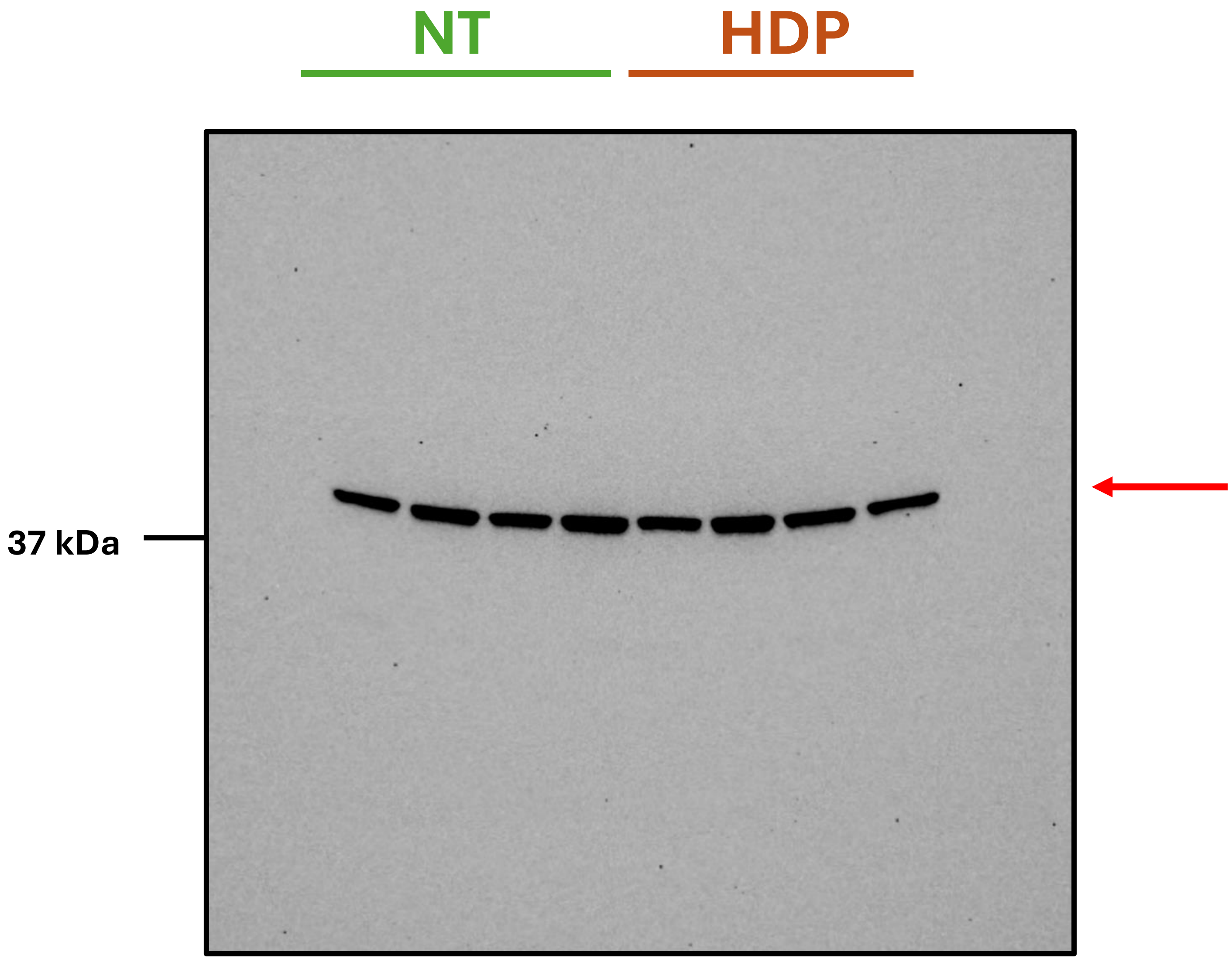

Figure 2A:
NRF2
16+5 hr
JEG3 Cells

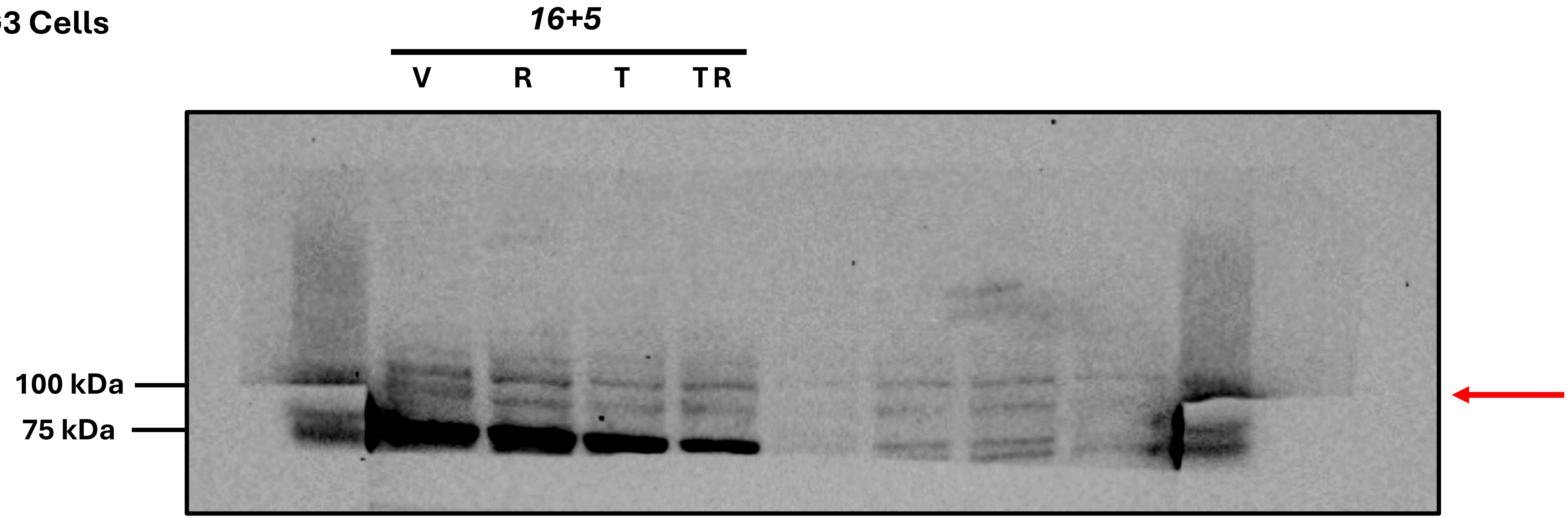

Figure 2A:
HDAC1
16+5 hr
JEG3 Cells

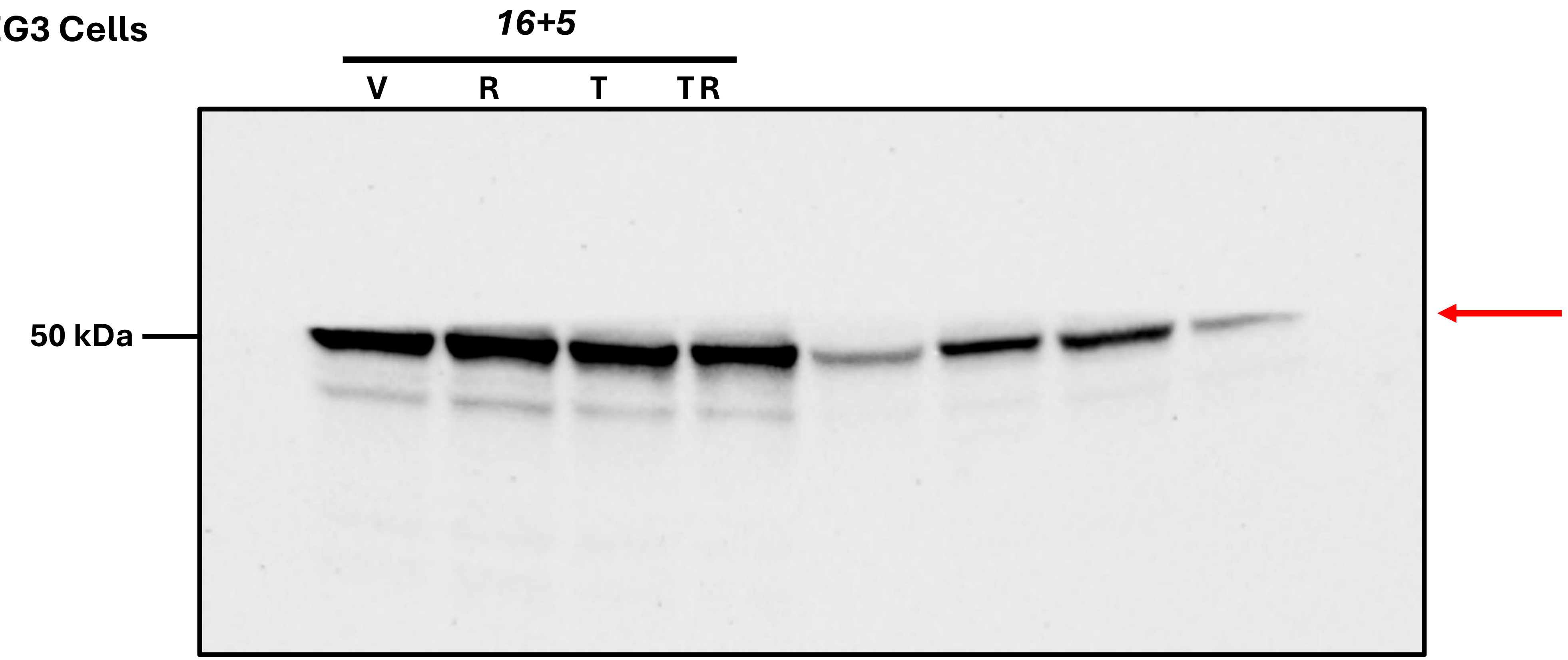

* This blot/image was used in figure 7A

Figure 2A:
NRF2
16+10 hr
JEG3 Cells

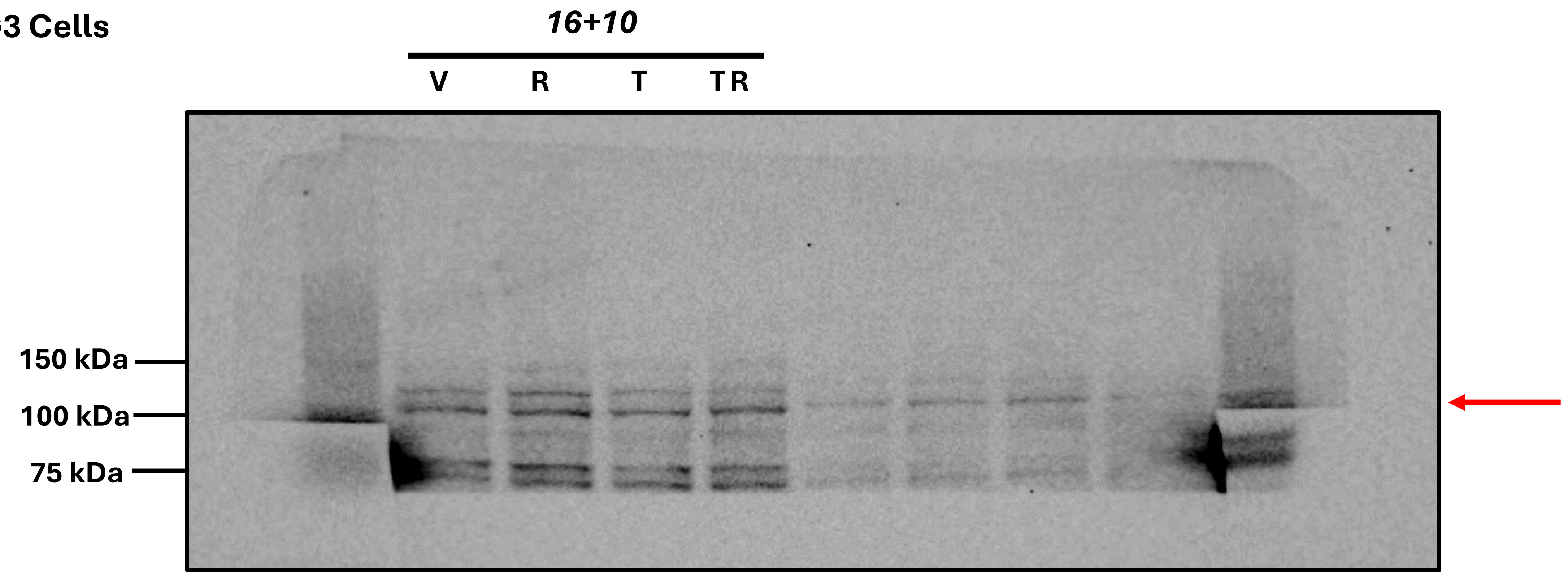

Figure 2A:
B-actin
16+10 hr
JEG3 Cells

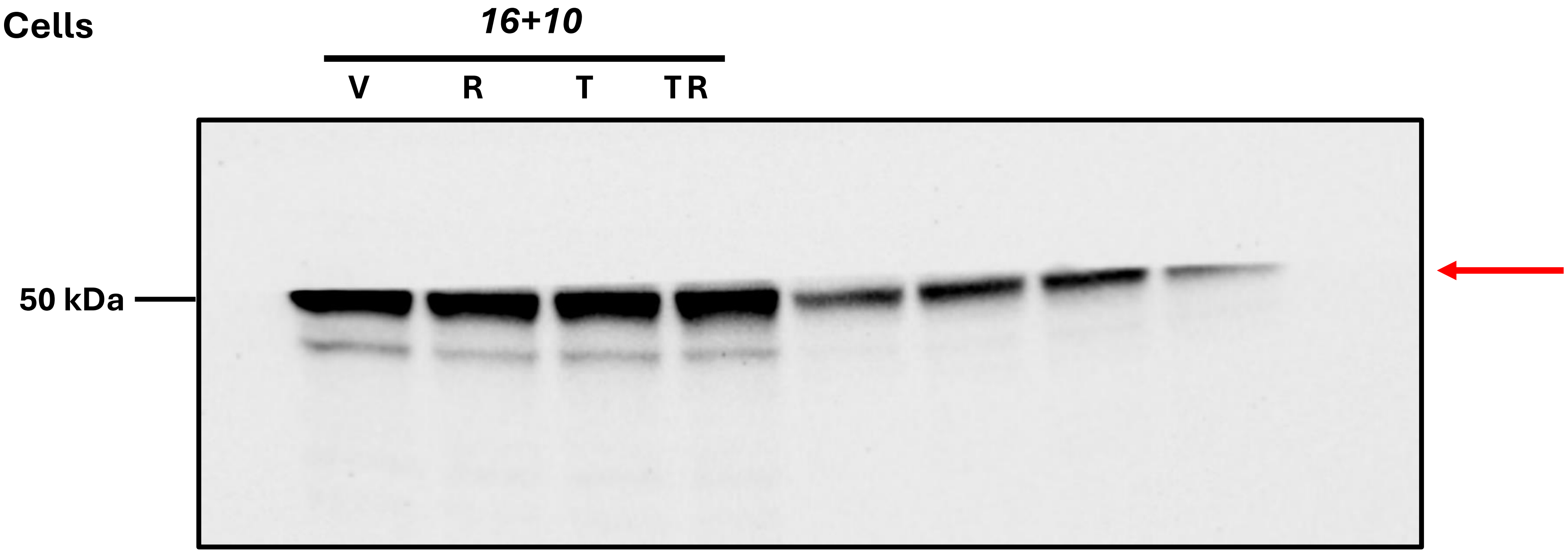

* This blot/image was used in figure 7A

Figure 5I:
OXPHOS
JEG3 Cells

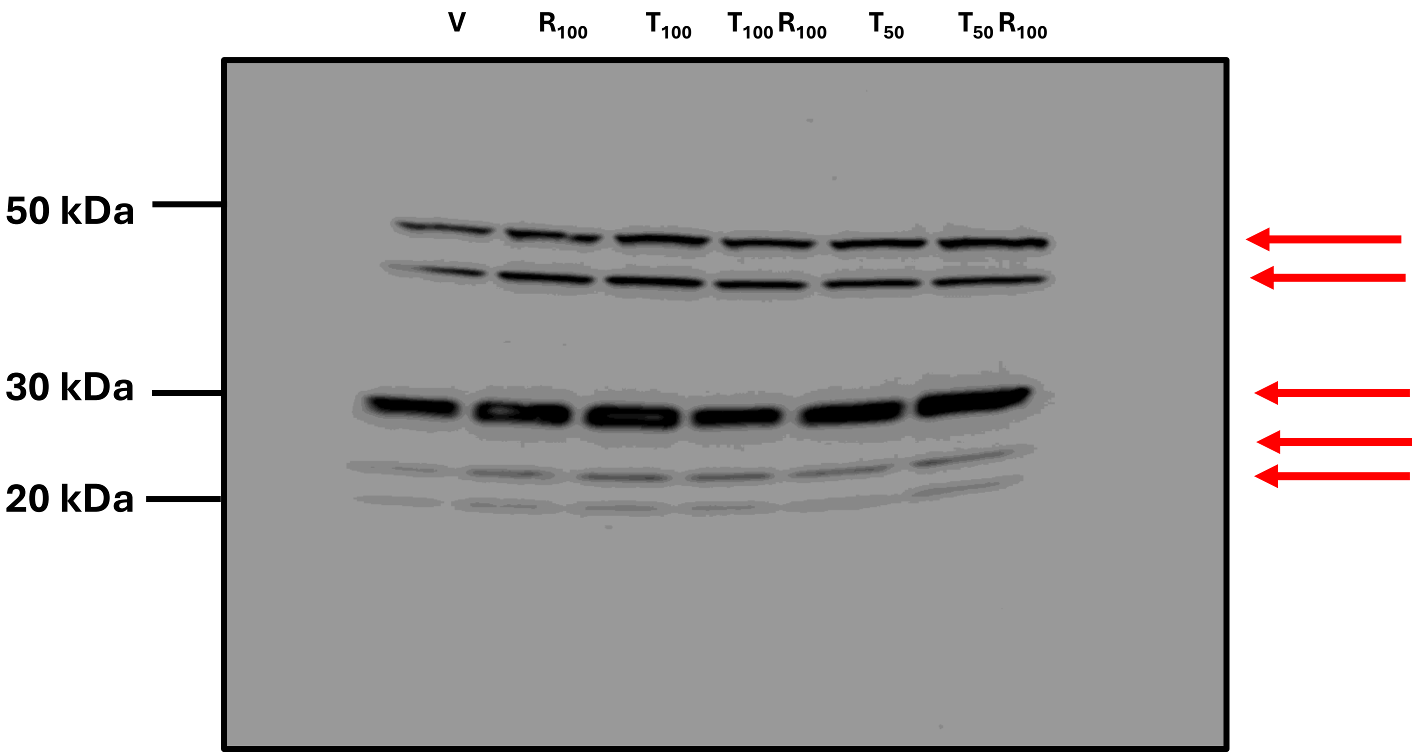

Figure 5I:
VDAC
JEG3 Cells

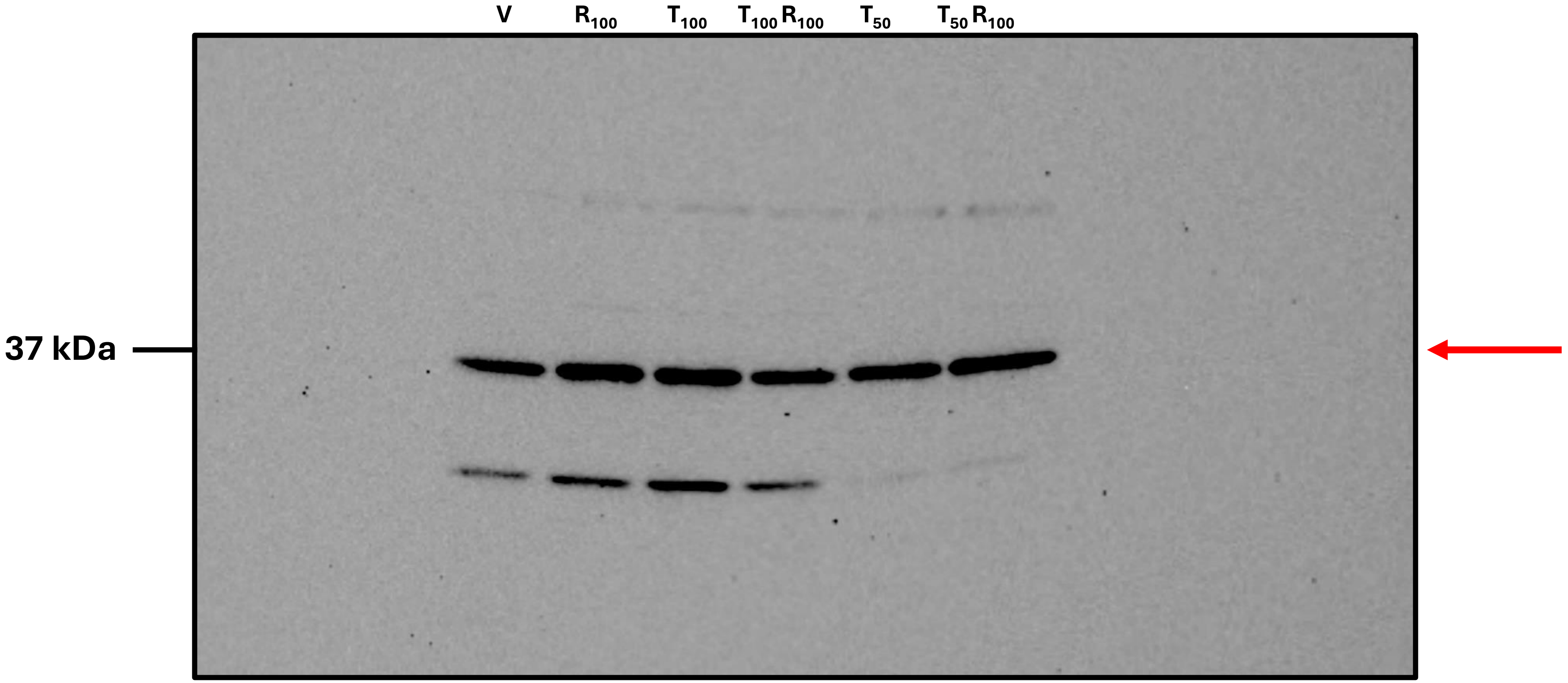

Figure 7A:
PGC1a
JEG3 Cells

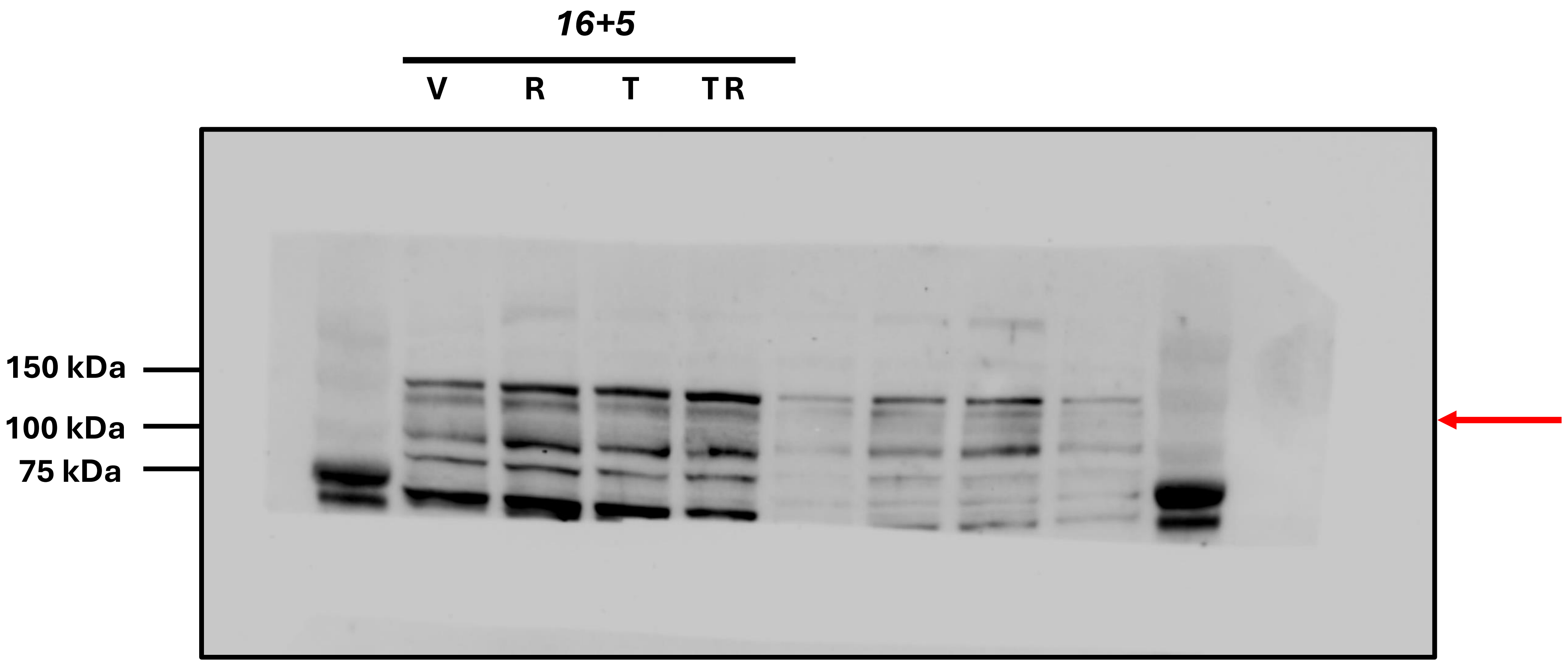

Figure 7A:
HDAC1
16+5 hr
JEG3 Cells

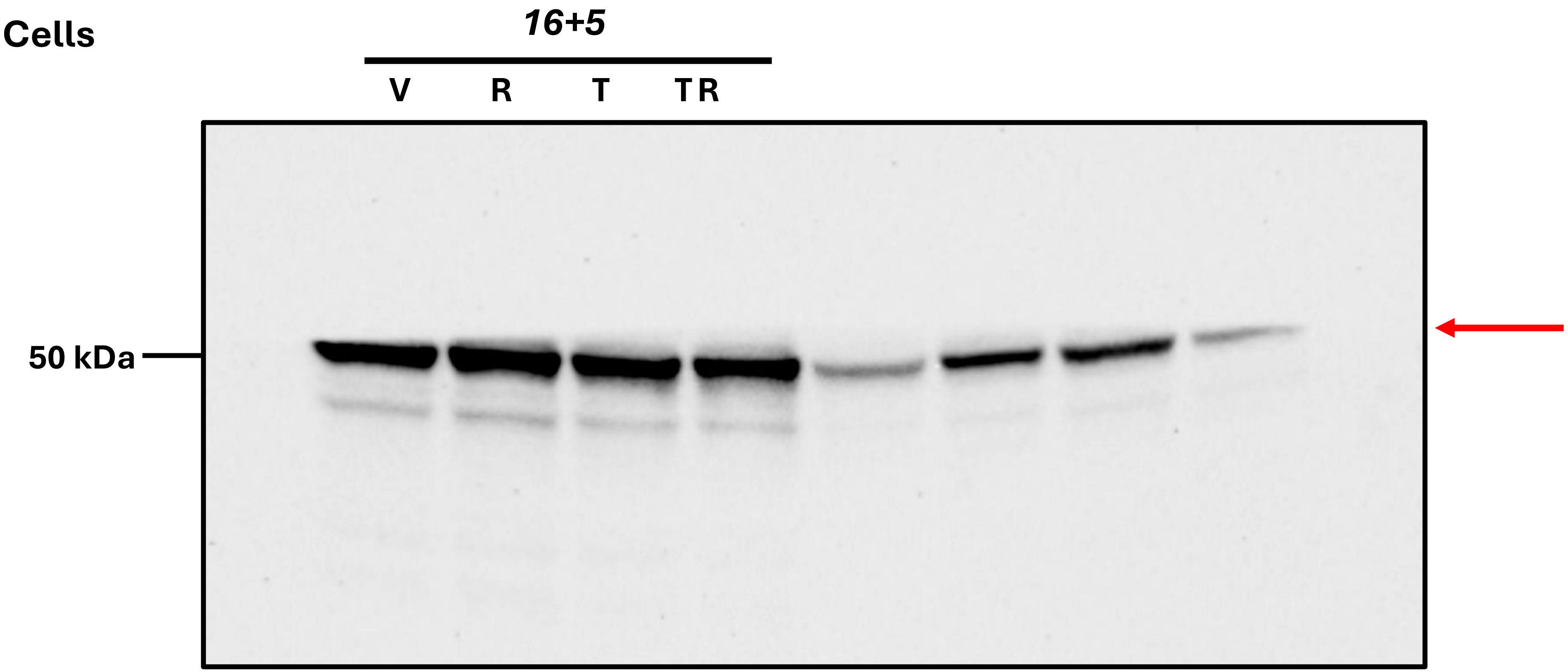

* This blot/image was used in figure 2A

Figure 7A:
PGC1a
JEG3 Cells

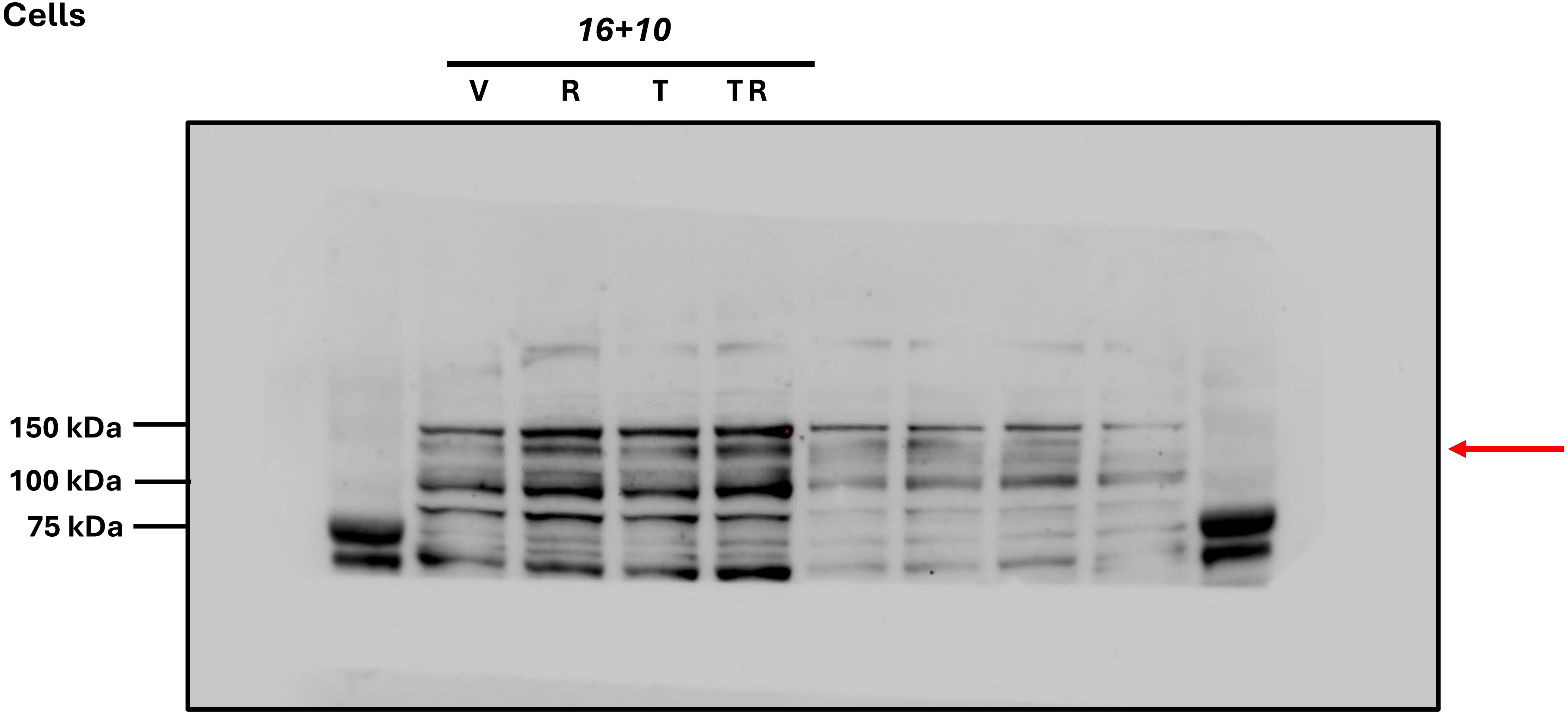

Figure 7A:
B-actin
16+10 hr
JEG3 Cells

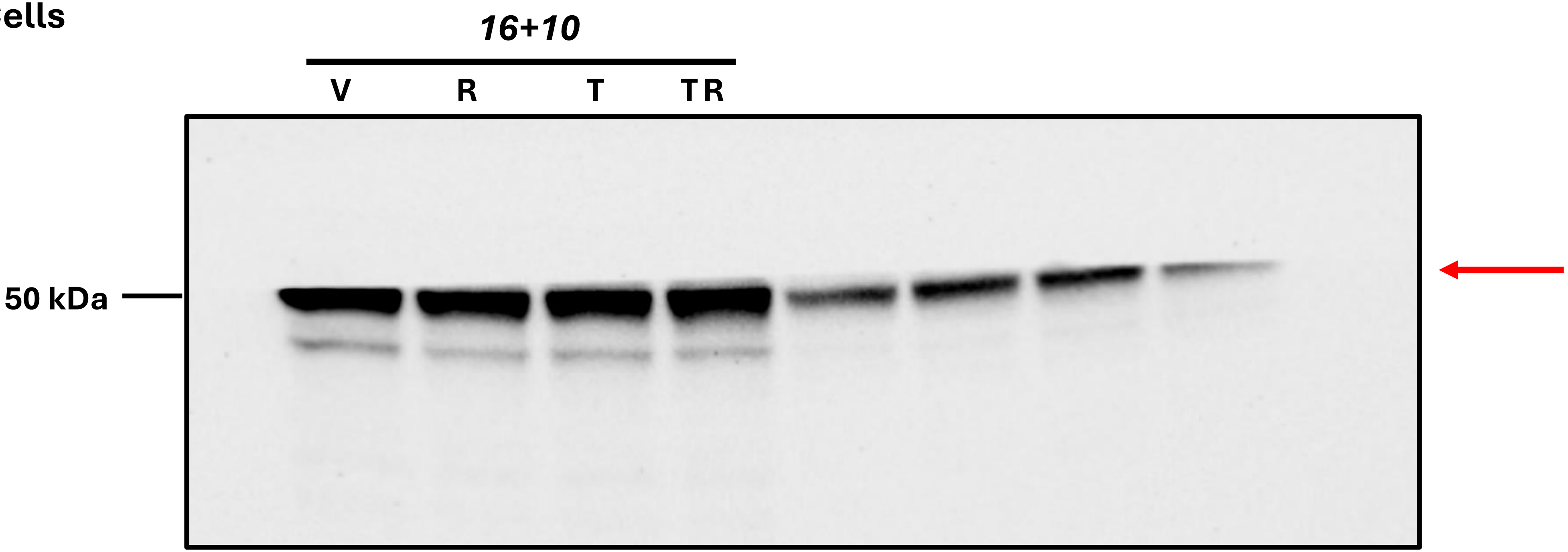

* This blot was used in figure 2A
