# Supplementary material for: RvD2 mitigates TNFɑ-Induced mitochondrial reactive oxygen species through NRF2 signaling in placental trophoblasts
Source: Front Physiol. 2025 Apr 2;16:1547940. doi: 10.3389/fphys.2025.1547940 (PMC12000658; doi:10.3389/fphys.2025.1547940)
Supplement: Supplementary file 2 [file DataSheet1.pdf]

RvD2 Mitigates TNFα-Induced Mitochondrial Reactive Oxygen Species Through NRF2 Signaling in Placental Trophoblasts

Taija Hahka[1,2,3*], Deekshika Sekar[3], Prakash Kumar Sahoo[4], Aiswariya Ravi[3], Colman Freel[1,2], Chandan Krishnamoorthy[3], Sankar Ramamurthy[3], Rebekah Rapoza[2], Rebecca Drakowski[2], Anum Akbar[2], Matt VanOrmer[2], Melissa Thoene[2], Corrine K. Hanson[5], Tara Nordgren[6], Sathish Kumar Natarajan[3,5*], Ann Anderson Berry[1,2]

[1]*Department of Cellular and Integrative Physiology, University of Nebraska Medical Center; Omaha, Nebraska*
[2]*Department of Pediatrics, University of Nebraska Medical Center; Omaha, Nebraska*
[3]*Department of Nutrition & Health Sciences, University of Nebraska at Lincoln; Lincoln, Nebraska*
[4]*Department of Microbiology & Immunology, Indiana University School of Medicine; Indianapolis, IN*
[5]*College of Allied Health Professions, University of Nebraska Medical Center; Omaha, Nebraska*
[6]*Department of Environmental and Radiological Health Sciences, Colorado State University; Fort Collins, Colorado*

Short Title: RvD2 attenuates redox signaling

Corresponding author: Taija Hahka* *and* Dr. Sathish Kumar Natarajan*

*Address for Correspondence:
Taija Hahka *and* Sathish Kumar Natarajan, PhD
Department of Cellular and Integrative Physiology *and* Department of Nutrition & Health Sciences
University of Nebraska Medical Center *and* University of Nebraska-Lincoln
985850 Nebraska Medical Center *and* 229 Filley Hall, Lincoln, NE 68583-0806
Omaha, NE 68198-5850 *and* Lincoln, NE 68583-0806

Phone: 402-805-7520
Email: taija.hahka@unmc.edu and snatarajan2@unl.edu

Supplementary Figure 1:

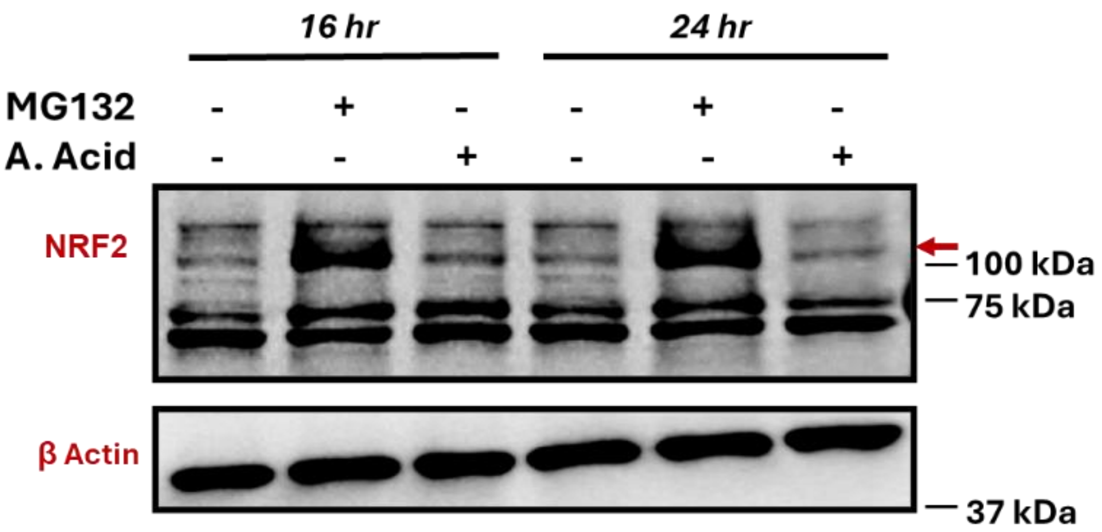

Supplementary Figure 1: MG-132 proteasome inhibitor upregulated NRF2 protein expression. A 16- and 24-hour treatment of JEG-3 cells with 12.5 μM of the proteasome inhibitor MG-132 resulted in upregulation of NRF2, with the changing band (red arrow) appearing at approximately 100-110 kDa. Ascorbic acid (A. Acid) at 1 mM did not alter the intensity of the NRF2 band.

Supplementary Figure 2:

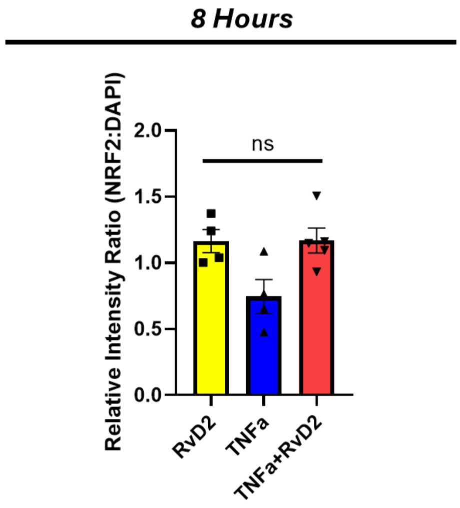

Supplementary Figure 2: Statistically significant changes in NRF2 expression were not observed at 8 hours of RvD2 and TNFα+RvD2 treatment. Quantified relative intensity of NRF2 expression from immunofluorescence images, converted to binary format and normalized to DAPI. 8 h treatment of RvD2 (100 nM), TNFα (50 ng/mL), and a combination of both treatments. Data presented as mean ± SEM. Comparing each group; ns, no significance; *n=4-5* per group.

Supplementary Figure 3:

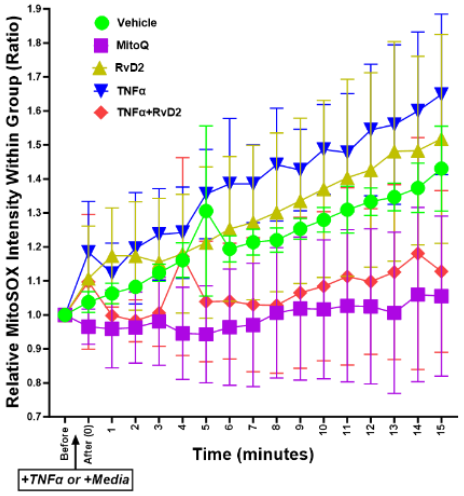

Supplementary Figure 3: Raw data of Relative MitoSOX intensity analysis compared within groups only (ratio taken from initial image set to one and compared to each sequential image). This is the complete set of data from **Figure 4A** showing the ratio of Before: After image intensity for each minute graphed for each group. This data set is not normalized to the Vehicle for that data instead depicts the ratio of Before: After image intensity.

Supplementary Figure 4:

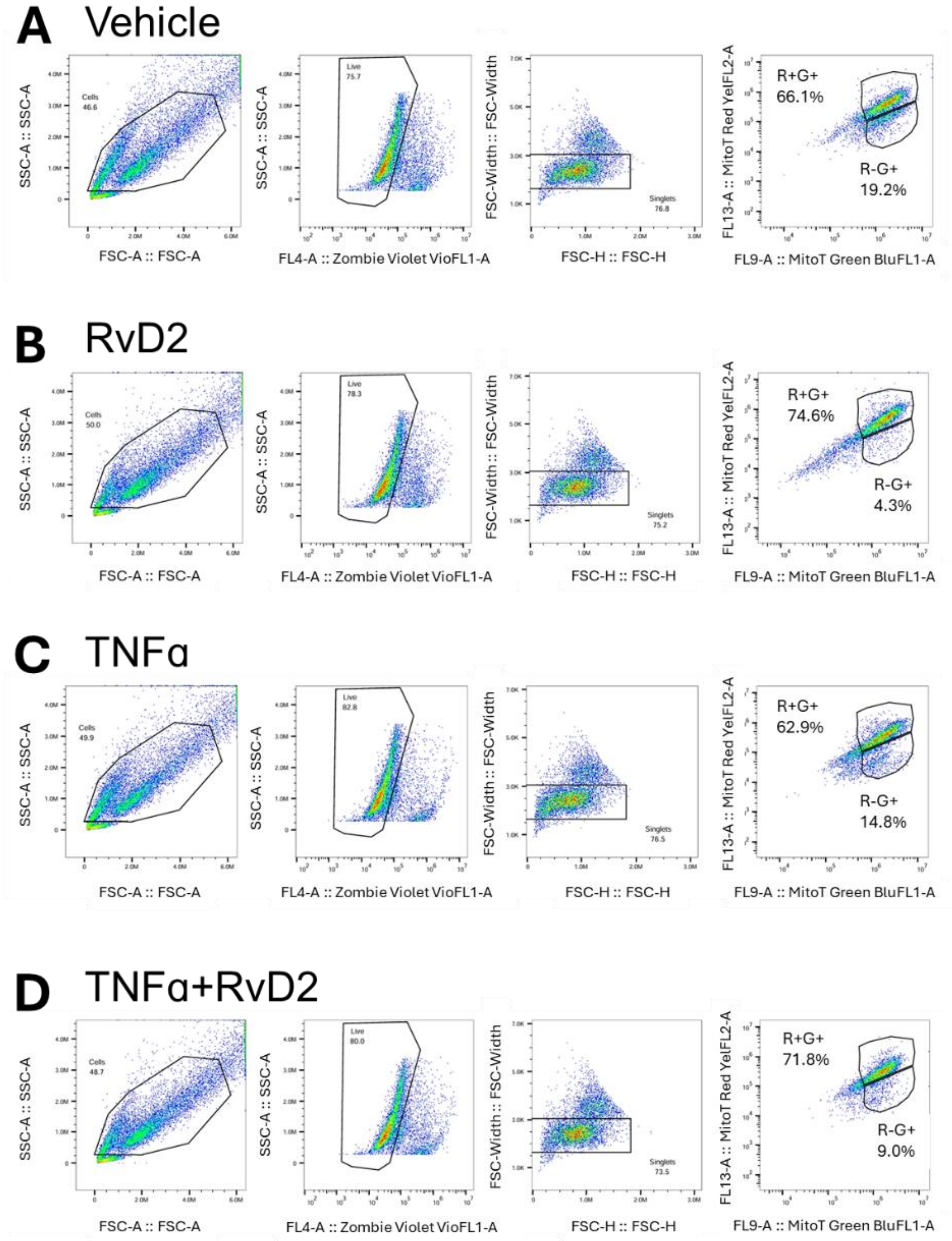

Supplementary Figure 4: Gating strategy for each treatment group. JEG-3 cells were pretreated with 100 nM of RvD2 for 16 h, followed by a 1-hour exposure to 100 ng/mL of TNFα. Cells with active mitochondria were classified as MitoTracker™ Red $^{high\ (+)}$ and MitoTracker™ Green $^{high\ (+)}$, denoted as "R+G+". Cells with inactive mitochondria were classified as MitoTracker™ Red $^{low\ (-)}$ and MitoTracker™ Green $^{high\ (+)}$, denoted as "R-G+". **A)** Vehicle, **B)** RvD2, **C)** TNFα, **D)** TNFα+RvD2 treatment groups.
